# Gingiva-Derived Mesenchymal Stem Cells: Potential Application in Tissue Engineering and Regenerative Medicine - A Comprehensive Review

**DOI:** 10.3389/fimmu.2021.667221

**Published:** 2021-04-16

**Authors:** Dane Kim, Alisa E. Lee, Qilin Xu, Qunzhou Zhang, Anh D. Le

**Affiliations:** ^1^Department of Oral & Maxillofacial Surgery & Pharmacology, School of Dental Medicine, University of Pennsylvania, Philadelphia, PA, United States; ^2^Center of Innovation & Precision Dentistry, School of Dental Medicine, School of Engineering and Applied Sciences, University of Pennsylvania, Philadelphia, PA, United States; ^3^Department of Oral & Maxillofacial Surgery, Penn Medicine Hospital of the University of Pennsylvania, Philadelphia, PA, United States

**Keywords:** gingiva-derived mesenchymal stem cells, neural crest, immunomodulation, anti-inflammation, regenerative therapy

## Abstract

A unique subpopulation of mesenchymal stem cells (MSCs) has been isolated and characterized from human gingival tissues (GMSCs). Similar to MSCs derived from other sources of tissues, e.g. bone marrow, adipose or umbilical cord, GMSCs also possess multipotent differentiation capacities and potent immunomodulatory effects on both innate and adaptive immune cells through the secretion of various types of bioactive factors with immunosuppressive and anti-inflammatory functions. Uniquely, GMSCs are highly proliferative and have the propensity to differentiate into neural cell lineages due to the neural crest-origin. These properties have endowed GMSCs with potent regenerative and therapeutic potentials in various preclinical models of human disorders, particularly, some inflammatory and autoimmune diseases, skin diseases, oral and maxillofacial disorders, and peripheral nerve injuries. All types of cells release extracellular vesicles (EVs), including exosomes, that play critical roles in cell-cell communication through their cargos containing a variety of bioactive molecules, such as proteins, nucleic acids, and lipids. Like EVs released by other sources of MSCs, GMSC-derived EVs have been shown to possess similar biological functions and therapeutic effects on several preclinical diseases models as GMSCs, thus representing a promising cell-free platform for regenerative therapy. Taken together, due to the easily accessibility and less morbidity of harvesting gingival tissues as well as the potent immunomodulatory and anti-inflammatory functions, GMSCs represent a unique source of MSCs of a neural crest-origin for potential application in tissue engineering and regenerative therapy.

## Introduction

Mesenchymal stromal cells (MSCs) represent a heterogeneous population of postnatal stem cells with self-renewal, multipotent differentiation, and immunomodulatory capabilities (1). Due to the heterogeneity, the International Society of Cell & Gene Therapy (ISCT) initially defined human MSCs based on three minimal criteria, including: 1) the plastic plate adherence; 2) the expression of a panel of cell surface markers such as CD73, CD90, and CD105 but negative for hematopoietic cell markers such as CD34, CD45, CD11b, CD14, CD19, and human leukocyte antigen-D related (HLA-DR or HMC-II) surface molecules; 3) the trilineage (osteogenic, adipogenic, and chondrogenic) differentiation potentials (2). In the last two decades, substantial preclinical and clinical studies have demonstrated the critical role of MSCs in tissue homeostasis and their potential application in tissue engineering and regenerative medicine (TE/RM) as a cell-based regenerative therapy for treating a large spectrum of autoimmune and inflammatory diseases and regenerating damaged tissues (3–5). Originally, it was proposed that MSCs exert their regenerative therapeutic effects as replacement cells due to their multipotent capacities, but a growing body of evidence supports the novel notion that MSC-mediated therapeutic effects are coined by their potent immunomodulatory/anti-inflammatory functions and pleiotropic properties conferred by their secretomes containing a myriad of biologic factors such as extracellular vesicle (EV)-dependent- and/or independent-growth factors, cytokines, hormones, miRNAs, and other bioactive soluble factors (5, 6). In 2019, ISCT included extra criteria to define MSCs, including their tissue origin and robust matrix of functional assays, including quantitative RNA analyses of selected genes, flow cytometry of cell surface markers and protein analysis of MSC secretome (7).

MSCs have been isolated from various adult and neonatal tissues, including bone marrow, adipose tissue, skin, umbilical cord, Wharton’s jelly, placenta, and dental tissues (5, 8, 9). MSCs derived from different anatomical tissues may be of different developmental origins and have significantly different transcriptomic signatures and biological properties and functions, e.g. the multipotent differentiation and proliferation potentials, cellular senescence, secretome & immunomodulatory functions (4). Therefore, the source of MSCs constitutes one of the major factors that contribute to the considerable variation in their regenerative therapeutic potentials. In recent years, adult MSCs derived from dental tissues have attracted more and more attention in the field of regenerative medicine due to their unique developmental origin and a relatively faster proliferation rate, and genomic stability compared to MSCs of other tissue origins (10, 11). In 2000, the first type of MSCs was isolated from human dental pulp tissues (DPSC) (12). Since then, MSCs have been isolated from various dental tissues, including exfoliated deciduous teeth (SHED) (13), periodontal ligament (PDLSC) (14), gingiva (GMSC) (15), apical papilla (SCAP), dental follicle (DFSC), and tooth germ stem cells (TGSC) (11). Similar to other sources of MSCs, particularly those derived from the bone marrow and adipose tissues, the dental MSCs possess potent self-renewal, multipotent differentiation, and immunomodulatory/anti-inflammatory properties, thus making them a promising alternative source of MSCs applicable in tissue engineering and regenerative medicine (8, 9, 11).

The human gingiva is a unique masticatory keratinized mucosal tissue and an essential component of the periodontal apparatus characterized by its rapid wound healing property with minimal scar-formation (16). Similar to most craniofacial tissues, the connective tissue of the gingiva develops from both the neural crest and the mesenchyme (17). Since its initial isolation and characterization in 2009 (15), accumulating studies have demonstrated the promising potential of GMSCs as a readily accessible and expandable source of MSCs that can be easily obtained through minimally invasive surgical techniques (18–20). In the present review, we focus on updating the progress in the study of GMSCs, particularly, their unique properties and biological functions, and highlighting their potential application in tissue engineering and regenerative therapy of a variety of diseases.

## Isolation and Characterization of Human Gingiva-Derived Mesenchymal Stem Cells

**Figure 1:**
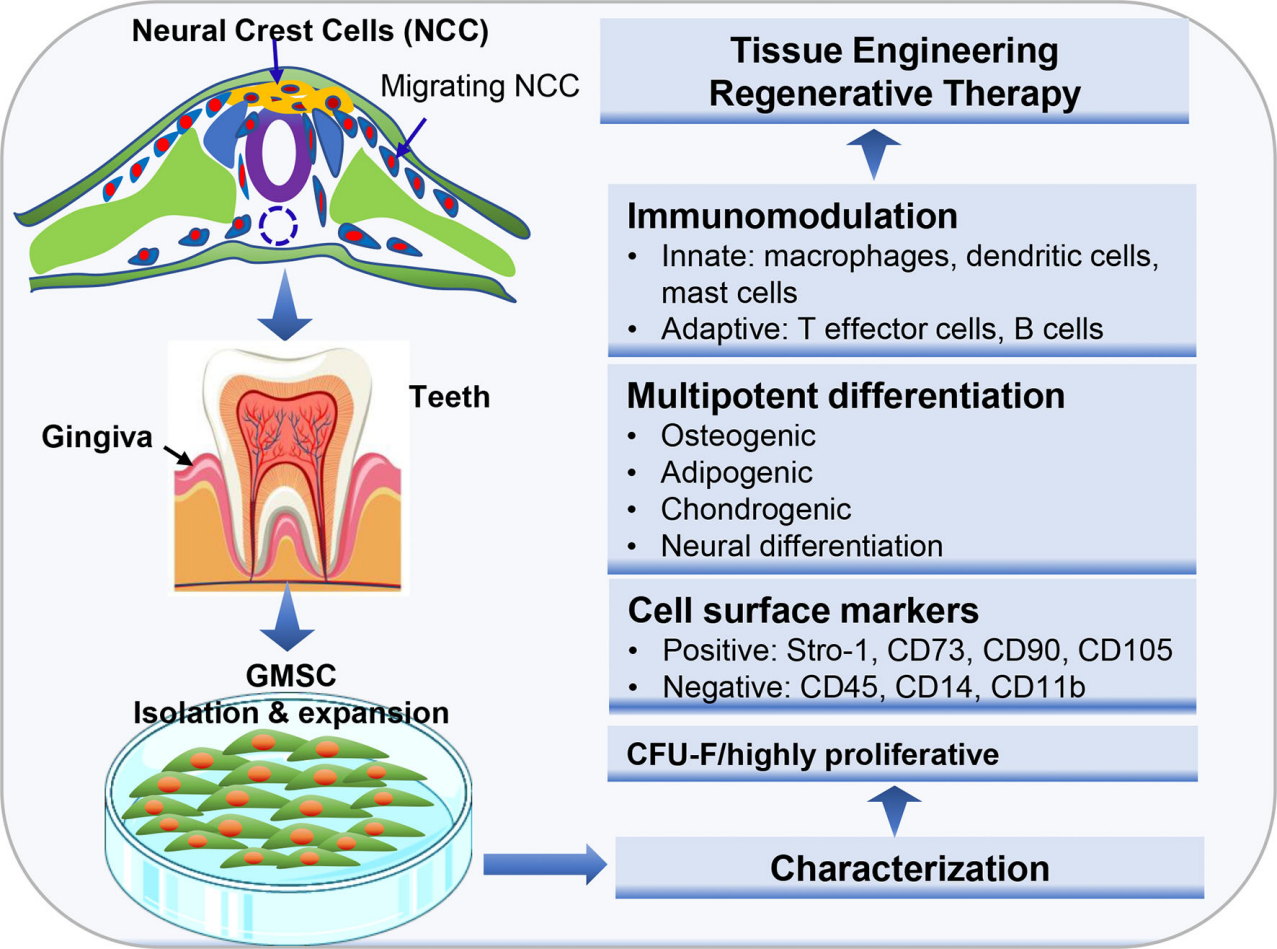
Isolation and characterization of mesenchymal stem cells derived from gingival tissues of neural crest origin. A unique subpopulation of mesenchymal stem cells can be isolated from neural crest-derived gingival tissues (GMSC), thus representing a reservoir of neural crest-derived MSCs. CFU-F, colony forming unit-fibroblast. Portions of this figure were made using templates from SMART SERVIER MEDICAL ART (https://smart.servier.com) and Vecteezy (https://www.vecteezy.com).

Histologically, gingival tissues are composed of the top layer of keratinocytes, the basal cell layer, and the submucosal spinous lamina propria/connective tissues. Initially, it was found that human gingival tissues harbor clustered immunostaining signals for stem cell-related genes such as Oct-4, SSEA-4, and STRO-1 in the lamina propria/connective tissue layers, suggesting the existence of a subpopulation of adult stem cells in human gingival tissues (15, 21). Subsequently, a unique population of progenitor cells was isolated from normal human gingival tissues by our group, which are characterized by their fibroblast-like spindle morphology, the colony forming unit-fibroblast (CFU-F), the expression of a panel of MSC-related cell surface markers such as CD73, CD90, CD105, SSEA-4, and STRO-1 but negative for hematopoietic cell markers like CD34 and CD45, and the multipotent differentiation into osteocytes, adipocytes, and neural types of cells (15). Later on, a similar population of progenitor cells was isolated and characterized from human gingival tissues by several other groups (**Table 1**) (21–27). In addition, these cells have been shown to possess the capability to generate both connective and bone-like tissues following ectopic subcutaneous transplantation into immunocompromised NOD/SCID mice (15, 21, 26, 27, 29). Taken together, the properties of these progenitor cells derived from human gingival tissues fulfill the minimal criteria of ISCT to define mesenchymal stromal/stem cells, thus designated as gingiva-derived mesenchymal stem/stromal cells (GMSCs) (**Figure 1**).

**Table 1:** Isolation and characterization of human GMSCs.

Isolation method	Cell surface markers	Multipotency	Refs
Enzymatic	CD73, CD90, CD105, SSEA4, STRO-1, CD146	Osteocytes, adipocytes, neural cells, endothelial cells	Zhang et al. (15)
Enzymatic	CD29, CD44, CD90, CD73	Osteocytes, adipocytes, chondrocytes	Tomar et al. (22)
Explant	CD29, CD44, CD73, CD90, CD105, STRO-1	Osteocytes, adipocytes, chondrocytes	Fournier et al. (23)
Explant	CD13, CD44, CD73, CD90, CD105	Osteocytes, adipocytes, chondrocytes	Mitrano et al. (24)
Enzymatic	CD29, CD44, CD90, CD105, CD146, STRO-1	Osteocytes, adipocytes, chondrocytes	Tang et al. (21)
Enzymatic	CD29, CD90, CD105, STRO-1	Osteocytes, adipocytes, chondrocytes	Wang et al. (25)
Enzymatic	CD44, CD73, CD90, CD105, CD166	Osteocytes, adipocytes, chondrocytes	Ge et al. (26)
Enzymatic	CD29, CD90, CD105, CD146, STRO-1	Osteocytes, adipocytes, chondrocytes	Yang et al. (27)
Enzymatic	CD29, CD90, CD105, CD146, STRO-1	Osteocytes, adipocytes, odontogenic	Gao et al. (28)

Despite the similar properties shared with MSCs of other tissue origins, GMSCs possess some unique properties, particularly their relatively high proliferative potentials. Several lines of evidence have shown that GMSCs exhibited higher proliferation capacity or less population doubling time than bone marrow-derived MSCs (BMSCs) (15, 22, 30) and umbilical cord-derived MSCs (31). For instance, Tomar et al. reported that human GMSCs has a mean population doubling time of 39.6±3.2h, which is much less than that of BMSCs (80.4±1.2h) (22). Meanwhile, they found that GMSCs display stable morphology and do not lose MSC characteristics and maintain telomerase activity following long-term cultures (22). In comparison to other types of dental MSCs, previous studies indicated that GMSCs possess less osteogenic potentials than PDLSCs (27, 32, 33). However, compared to PDLSCs, GMSCs are less susceptible to replicative senescence and pro-inflammatory cytokine-induced impairment in osteogenic potentials *in vitro* and ectopic bone formation *in vivo (*27). In term of their multipotency, GMSCs not only possess the mesodermal trilineage differentiation potentials (osteocytes, adipocytes, and chondrocytes), but also the potential to transdifferentiate into ectodermal and endodermal cell lineages, such as neural cells (15, 34), keratinocytes (35), and endothelial cells (15, 36), and odontogenic cells (28), under defined induction conditions, respectively. Due to these compelling characteristics, GMSCs represent a promising source of adult MSCs with potent regenerative potentials (**Figure 1**).

## Gingiva: A Reservoir of Adult Neural Crest-Derived MSCs

Neural crest (NC) is formed at the neural plate border and composed of a unique population of multipotent stem cells that arise during vertebrate embryonic development (37). The transient premigratory neural crest cells (NCCs) delaminate from the neural tube, undergo epithelial-mesenchymal transition, transit to migratory NCCs which migrate to various locations and give rise to more than thirty different types of ectodermal, mesodermal, and endodermal derivatives, including craniofacial skeleton, peripheral and enteric nervous system, pigment and some endocrine cells, and many other cell types, e.g. mesenchymal cells, throughout the body (37, 38). Interestingly, recent preclinical studies in animal models have demonstrated the existence of reservoirs of NC-derived multipotent stem cells in various neonatal and postnatal tissues, including bone marrow, sciatic nerve, skin and hair follicle, palatal tissue, and so on [extensively reviewed elsewhere (38, 39)]. Due to their wide existence throughout adulthood and their high stem cell potency, adult NC-derived stem cells may represent a promising source of stem cells in tissue engineering and regenerative medicine.

Developmentally, most cranial/dental tissues are derived from neural crest (37, 38). A unique subpopulation of multipotent mesenchymal stem cells with NC-derived stem cell properties have been isolated from various adult human dental tissues, including dental pulp (DPSCs) (12, 40), oral mucosa and gingiva (41–44), apical papilla (APSC) (45), dental follicle (DFSC) (46), and periodontal ligament (PDLSC) (47). These studies suggest that dental tissues serve as important reservoirs of NC-derived multipotent stem cells. Regarding the NC-derived stem cells in adult human gingival tissues, early studies indicated that adult human oral mucosal/gingival lamina propria harbors a subpopulation of MSCs endowed with NC-derived stem cell properties (41, 44), such as increased colony-forming efficiency (CFE) in the presence of Jagged 1 (a NOTCH ligand), the expression of a number of neural crest markers within the developing colonies *in vitro*, and the multipotent differentiation potentials into both mesodermal (chondrogenic, osteoblastic, and adipogenic) and ectodermal (neuron and Schwann-like cells) cell lineages (44). When cultured on chitosan membranes, human gingival mesenchymal stromal cells spontaneously form 3D-spheroids with enriched expression of neural crest stem cell (NCSC)-related genes, e.g. SOX10 and SLUG, and increased neuronal and chondrogenic differentiation potentials (48, 49). A recent study showed that human gingival mesenchymal cells expressed NC-related genes Nestin, Snai1, Twist1, Pax3, Sox9 and FoxD3, and generated neurospheres with significantly increased expression of NC-related genes and down regulation of fibroblast-related type I collagen (43). Altogether, these findings suggest that adult human gingival tissues might be a potential reservoir of NC-derived multipotent stem cells (**Figure 1**).

To date, several lines of evidence have demonstrated the NC-origin of adult gingival mesenchymal stem cells by employing different NC-reporter mouse models. Using a Lgr5 reporter mice, Boddupally et al. reported that the tongue and certain areas of oral mucosa harbor a subpopulation of Lgr5^+^ stromal stem cells, which are derived from embryonic NC and display properties of neural crest stem cells (NCSC) with potent self-renewal and multipotent differentiation capacities. They express high levels of neural crest-associated genes, such as Sox9, Twist1, Snail, Myc, Ets1, Crabp1, Epha2, and Itgb1,and are maintained not only during embryonic development but also postnatally (50). Using *Wnt1-Cre;R26R* and *Wnt1-Cre;ZsGreen* double transgenic mouse models, Xu X et al. reported that about 90% of GMSCs are derived from cranial NCC while only about 10% of them from the mesoderm, whereas NC-derived GMSCs have greater potentials to differentiate into neural cells and chondrocytes than mesoderm-derived GMSCs (51). Using NC-reporter Wnt1-Cre/R26RYFP mice, Fournier et al. showed that NC-derived cells are retained in the gingival connective tissue of aged mice (43). Most recently, using NC-reporter Pax3-cre::Rosa^tomato^ and Wnt1-creER^T^::Rosa^tomato^ transgenic mice, Isaac et al. have clarified that more than 85% of the connective cells in the palate, palatal gingiva, and vestibular mandibular gingiva are derived from the neural crest, while 65% are in the buccal mucosa, which may contribute to the scarless oral wound healing process (17). Meanwhile, the *in vitro* studies showed that these NC-derived gingival mesenchymal stem cells are endowed with multipotential properties as well as a specific migratory and contractile phenotype compared to those mesoderm-derived stromal cells such as abdominal dermis fibroblasts (17). Taken together, these findings further support the notion that adult gingival tissue is an easily accessible reservoir of NC-derived MSCs.

## Immunomodulatory and Anti-Inflammatory Functions of GMSCs

**Figure 2:**
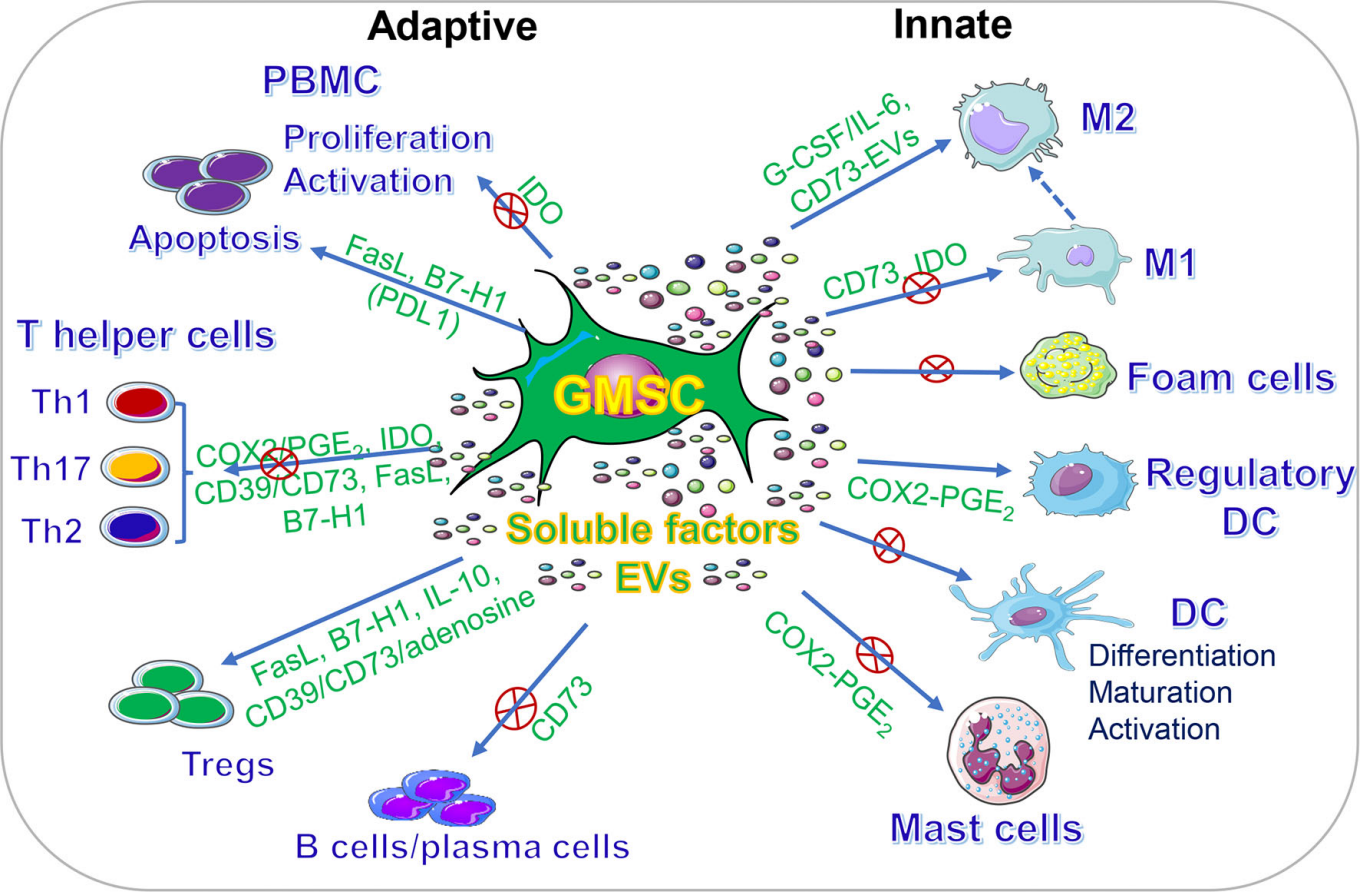
Immunomodulatory effects of GMSCs on both innate and adaptive immune cells. PBMC, peripheral blood mononuclear cells; M2, M2 macrophages; M1, M1 macrophages; DC, dendritic cells; IDO, indoleamine 2,3-dioxygenase; COX2, cyclooxygenase-2; PGE2, prostaglandin 2; FasL, Fas ligand; EVs, extracellular vesicles; IL-10, interleukin-10; IL-6, interleukin-6. “⊕” means blocking or inhibiting. Portions of this figure were made using templates from SMART SERVIER MEDICAL ART (https://smart.servier.com).

In addition to their multipotent differentiation capacities, GMSCs also possess potent immunomodulatory and anti-inflammatory functions through modulating the phenotype and activation of a variety of innate and adaptive immune cells both *in vitro* and *in vivo* (**Figure 2**, **Table 2**).

**Table 2:** Immunomodulation of GMSCs.

*In vitro*	*In vivo*	Mechanisms	Disease models	Refs
M1, M2 MФ, Th17	M1/M2 MФ	IL-6/G-CSF	Mice, skin wound	Zhang et al. (52)
	M2		Cutaneous radiation syndrome	Linard et al. (53)
Monocytes/macrophages, M1	Monocytes, Macrophages, Th1, Th2, Th17	CD73, IDO	AopE^−/−^ mice, Atherosclerosis	Zhang et al. (54)
M1/M2 macrophages	M1		Mice, periodontitis associated with hyperlipidemia.	Hong et al. (55)
Mast cells, Dendritic cells	Mast cells, CD8 T cells, Th17	COX2/PGE2	Mice, skin contact hypersensitivity (CHS)	Su et al. (56)
PBMC, Tregs (m)			GvHD	Tang et al. (21)
PBMC				Mitrano et al. (24)
	NK1.1^+^, CD11b^+^, total CD4 T cells, Tregs	IL-10/IL-10R	Mice, colitis	Lu et al. (57)
PBMC	Th1, Th17, Tregs	IDO	Mice, colitis	Zhang et al. (15)
T cell apoptosis (m)	Th17, Tregs	FasL	Mice, colitis	Xu et al. (51)
CD4^+^T cell apoptosis, Th17, Tregs (m)	Th17, Tregs	H2S-FasL	Mice, colitis	Yang et al. (58)
T cell apoptosis, Th1, Th17, Tregs(m)	Th1, Th17, Tregs	Acetylsalicylic acid (ASA) -FasL	Mice, Colitis, periodontitis	Yu et al. (59)
PBMC, apoptosis		FasL	Mice, skin wound	Jiang et al. (60)
	Th1/Th2, Tregs	PGE2	Mice, skin contact hypersensitivity (CHS)	Li et al. (61)
CD4^+^T proliferation; Th1, Th2, Th17 (m)	CD4^+^CD39^+^FoxP3^+^ Tregs	CD39/CD73	Mice, arthritis	Chen et al. (62)
PBMC, T cell apoptosis (m)	Th1, Th17, Tregs	FasL	Mice, arthritis	Gu et al. (63)
	Th1, Th17	CD39/CD73 or adenosine	Mice, arthritis	Luo et al. (64)
T cell proliferation Th1, Th17(m)	B/plasma cells Th1, Th17 Tregs	B7-H1/PD-L1 IFN-γ primed	Mice, arthritis	Wu et al. (65)
CD3^+^T cell proliferation Th1, Th17 (h)	CD4^+^ T cells	CD39-CD73-adenosine-IDO axis	Mice, GvHD	Huang et al. (66)
Tregs, Th1, Th17 (m)	Th1, Th17, Tregs	CD39	Mice, GvHD	Ni et al. (67)
T cell proliferation	Th1, Th17, Tregs	CD39/CD73	Mice, T1DM	Zhang et al. (68)
CD3^+^, CD4^+^, CD8^+^ T cells, Tregs (h)		PD-L1, IL-10, PGE2		De la Rosa-Ruiz et al. (69)
	CD8^+^T, Th1, Th17		Aplastic anemia	Zhao et al. (70)
B cells	B cells, Th2, Th17, Tfh	CD39 (–)CD73(+)	Mice, SLE nephritis	Dang et al. (71)
	Th17, Tregs	CD39	Mice, osteoporosis	Wu et al. (72)

### GMSC-Mediated Immunomodulatory Effects on Innate Immune Cells

The innate immune system is the first line of host defense, which consists of several types of innate immune cells, such as monocytes/macrophages, dendritic cells, neutrophils, and natural killer (NK) cells (73). Several lines of evidence have shown that GMSCs possess potent immunomodulatory effects on innate immune cells, particularly macrophages, dendritic cells (DCs), and mast cells (74).

### Macrophages

Macrophages, either derived from the hematopoietic progenitors or from the yolk sac, exist in all tissues throughout the body and play essential roles not only in innate immune responses but also in development, tissue repair, and homeostasis, whereas phenotypic and functional alterations of macrophages contribute to the development and progression of various pathological conditions (75, 76). As professional phagocytic cells, macrophages are characterized by their distinct phenotypic and functional plasticity, making it a challenge to appropriately classify them. Traditionally, macrophages have been simply classified into pro-inflammatory M1 and anti-inflammatory M2 macrophages according to their phenotypes and secreted pro- or anti-inflammatory mediators (76). In the last decade, a wealth of evidence has demonstrated the potent immunomodulatory effects of MSCs on monocytic and tissue-resident macrophages both *in vitro* and in various preclinical disease models *in vivo*, and such a pivotal cross-talk between MSCs and macrophages has been extensively reviewed (77). Generally, MSCs of different tissue origins can potently suppress M1 macrophage polarization, while boosting M2 macrophage polarization as evidenced by a decrease in the expression of M1-related genes (e.g. CD86 and iNOS) and the secretion of pro-inflammatory cytokines (e.g. TNF-α, IL-6, IL-1β) but an increase in the expression of M2-related (e.g. CD206, CD163, arginase-1) and the secretion of anti-inflammatory cytokines (IL-10 and TGF-β) (77).

Similarly, GMSCs also display potent immunomodulatory effects on macrophages. Our previous study showed that GMSCs are capable of polarizing macrophages into the M2 phenotype characterized by upregulated expression of CD206, increased secretion of anti-inflammatory cytokine IL-10 and phagocytotic activity as determined by flow cytometry and ELISA, respectively (52). Meanwhile, co-culture with GMSCs simultaneously suppressing M1 macrophage polarization with reduced expressions of CD86 and DC-specific intercellular adhesion molecule-grabbing nonintegrin (DC-SIGN/CD209) and decreased secretion of pro-inflammatory cytokine TNF-α as determined by flow cytometry and ELISA (52). In a murine skin wound healing model, systemically transplanted GMSCs home to the injury site and significantly promote protein expressions of M2 macrophage-related genes, RELMα and arginase-1, in wound areas as determined by fluorescence staining and Western blot (52). In a cutaneous radiation syndrome model in mice, intradermal administration of gingival fibroblasts substantially reduced the expression of M1 macrophage markers, such as inducible nitric oxide synthase (iNOS), while increased the expression of arginase-1 compared with irradiated-untreated skin as determined by qRT-PCR. Meanwhile, immunostaining staining showed increased infiltration of CD163^+^ M2 macrophages compared with nonirradiated and irradiated-untreated skin (53). In a high fat diet-induced atherosclerosis model in apolipoprotein E knock out (ApoE^−/−^) mice, systemic infusion of human GMSCs led to a similar and significant decrease in the frequency of splenic and blood macrophages as well as a drop in the frequency of total macrophages and F4/80^+^CD16/32^+^ M1 macrophages in the draining lymph nodes of ApoE^−/−^ mice. Meanwhile, infusion of GMSCs significantly reduced the frequencies of CD11b^+^ monocytes, particularly, the pro-inflammatory subset of CD11b^+^Ly-6C^hi^, in spleen and peripheral blood (54) as determined by flow cytometry. *In vitro* studies indicate that co-culture with GMSCs suppress the expression of M1 macrophage markers e.g. CD86 and HLA-DR, increase the expression of M2 macrophage marker CD206, and inhibit ox-LDL-induced foam cell formation partially through CD73 and indoleamine-2,3-dioxygenase 1 (IDO) signaling pathways (54). Similarly, Hong R et al. reported that co-culture with GMSCs significantly reduced the expression of M1-related pro-inflammatory cytokines of TNF-α, IL-6, and IL-1β and M1 markers CD86 and HLA-DR, while moderately increased the expression of IL-10 and CD206 as determined by RT-PCR, flow cytometry, and ELISA, respectively (55). These studies suggest that GMSCs have potent immunomodulatory effects on the phenotype and activation of macrophages. However, further studies are necessary to explore the detailed mechanisms of action of GMSCs in regulating macrophage polarization under different pathological settings.

### Mast Cells

Mast cells (MCs) constitute an important arm of the innate immune system and are characterized by their abundant cytoplasmic granules (78, 79). Upon activation by a variety of environmental stimulators, they release an abundance of bioactive mediators *via* degranulation with diverse biological functions, such as amplification of inflammatory responses by recruiting inflammatory cells and facilitating adaptive immune responses, promoting vascular permeability, and regulating angiogenesis and fibrosis, etc. Dysregulation of MC functions can contribute to the initiation and progression of various pathological conditions or diseases such as allergic and anaphylactic reactions, abnormal wound healing, fibrosis, and malignancies (79). Several lines of evidence have shown that MSCs can also modulate MC activation and functions (80–83). An early study showed that murine BMSCs effectively suppressed MC degranulation, pro-inflammatory cytokine production, chemokinesis and chemotaxis when co-cultured with direct cell-cell contact *in vitro* and following *in vivo* administration in a model of passive cutaneous anaphylaxis and a peritoneal degranulation assay (83). In addition, human umbilical cord blood-derived mesenchymal stem cells (hUCB-MSCs) have been shown to ameliorate atopic dermatitis by inhibiting MC degranulation and release of pro-inflammatory cytokines (80, 81). In a previous study, we showed that human GMSCs reduce the expression of MC marker CD117 and suppress *de novo* synthesis of the major pro-inflammatory cytokine, TNF-α and IL-4, from activated human HMC-1 mast cells in a cell-cell contact-independent manner as determined by flow cytometry and ELISA (56). Also, *in vivo* administration of GMSCs significantly suppressed MC degranulation and attenuated chronic hypersensitivity (CHS) of mice skin (56). Mechanistically, elevation of cyclooxygenase-2 and prostaglandin E_2_ (COX2/PGE_2_) and TGF-β appears to play a critical role in MSC-mediated suppression of MC activation both *in vitro* and *in vivo (*56, 80–83). These findings have highlighted the immunomodulatory effects of MSCs, including GMSCs, on MC activation and their potential application in regenerative therapy for MC-driven inflammatory diseases.

### Dendritic Cells

Dendritic cells (DCs) are highly professional antigen presenting cells (APC) that play key roles in immune responses through bridging innate and adaptive immunity (84). DCs are susceptible to various extrinsic signals that can induce differentiation and maturation of DCs, thus leading to immune responses. However, certain signals can also induce tolerogenic or regulatory phenotypes and functions in DCs, thus leading to compromised immune responses (85). Deregulated functions of DCs, either hyperactivated or tolerogenic, can contribute to the pathogenesis of various disorders, such as some autoimmune diseases, GvHD, rejection of organ transplantation, and malignancies (84, 85). Numerous studies have demonstrated the potent immunomodulatory effects of MSCs on DCs at multiple levels, such as induction of a regulatory phenotype, inhibition of their differentiation/maturation and release of pro-inflammatory cytokines, and inhibition of their antigen presenting capacities (86). Our previous study showed that co-culture with human GMSCs significantly inhibits the expression of mature DC markers, CD11c and CD80, and the release of pro-inflammatory cytokine IL-12 as determined by flow cytometry and ELISA, whereby the production of prostaglandin E_2_ (PGE_2_) plays a critical role (56). Further studies are necessary to explore the potential regulatory effects of GMSCs on DC functions *in vivo* under different DC-related pathological conditions.

### GMSC-Mediated Immunomodulatory Effects on Adaptive Immune Cells

Adaptive immune system constitutes another arm of immunity and can be divided into two categories, cell- and antibody-mediated immune responses, which are carried out by distinct types of lymphocytes, T cells and B cells, respectively (87). Among T cells, CD4^+^ T helper cells play critical roles in adaptive immune responses and act as key regulators of host health and disease. Classically, specialized subsets of T helper cells, such as T helper type 1 (Th1), Th2, Th17, CD4^+^CD25^+^FoxP3^+^ regulatory T cells (Tregs), are generated from naïve CD4^+^ T cells upon activation through the stimulation by a milieu of lineage-specifying cytokines and transcriptional factors, among which Tregs counteract immune responses conferred by other types of T helper cells through the production of anti-inflammatory cytokines such as IL-10 and TGF-β, thus contributing to immune homeostasis (87). A wealth body of studies, both *in vitro* and *in vivo*, have demonstrated the potent immunomodulatory effects of MSCs of different tissue-origins on various types of adaptive immune cells, particularly, various subsets of T helper cells, under different pathological settings, which has been extensively reviewed elsewhere (6). In the last decade, numerous studies have also recognized the potent immunomodulatory effects of GMSCs on different subtypes of adaptive immune cells both *in vitro* and *in vivo* (**Table 2**, **Figure 2**).

#### Peripheral Blood Mononuclear Cells (PBMC)

Early studies showed that human GMSCs can potently suppress the proliferation and activation of human PBMC stimulated either by phytohemagglutinin (PHA) (15), ConA (60) or allogenic lymphocytes in mixed lymphocyte reactions (MLRs) (21, 24). Mechanistically, GMSCs suppress PHA-stimulated T lymphocyte proliferation and activation in a cell-cell contact-independent manner, apparently mediated *via* increased IDO expression in GMSCs in response to IFN-γ signals secreted by activated PBMCs (15).

#### CD3^+^/CD4^+^ T Cells

Previous studies showed that co-culture with human GMSCs in a trans-well system significantly suppressed the proliferation and secretion of cytokines, IFN-γ (Th1), IL-4 (Th2), and IL17 (Th17) in mouse splenic CD4^+^CD25^−^ cells in response to T cell receptor stimulation with anti‐CD3 mAb as determined by flow cytometry, which is dependent on CD39/CD73 signals on GMSCs (62, 68). A recent study showed that human GMSCs are able to suppress both human CD3^+^ and CD4^+^ T cells proliferation *in vitro*, whereby the CD39/CD73/adenosine and/or IDO signals in GMSCs play an important role (66). However, another study indicated that human GMSCs suppress proliferation and activation of CD4^+^ and CD8^+^ T cells stimulated with anti‐CD3/anti-CD8 mAbs, which correlates with a decreased production of IFN-γ and TNF-α and the upregulation of programmed death ligand 1 (PD-L1) in MSCs and cytotoxic T-cell-associated Ag-4 (CTLA-4) in T-cells, and simultaneously, an increased production of IL-10 and PGE2 in the co-cultures (69). Most recently, Wu et al. reported that IFN-γ-pretreated GMSCs exerted stronger suppression on mouse T cell proliferation and cytokine secretion than control GMSCs, while the B7-H1 (PD-L1) blocking antibody abolished IFN-γ-pretreated GMSC-mediated inhibitory effects on T cell activation (65). In addition, several lines of evidence have shown that co-culture with mouse GMSCs in direct cell-cell contact induces mouse T cell apoptosis, whereby the expression of FasL on GMSCs plays an essential role (51, 58, 59, 63). These findings suggest that GMSCs suppress T cell proliferation/activation and induce T cell apoptosis through different mechanisms, which might be due to differences in GMSCs and target T cells derived from different species.

#### T Helper Cells

The potent immunomodulatory effects of MSCs of different tissue origins on different subtypes of T helper cells, particularly Th1, Th2, and Th17 cells, have been well documented (6). Similarly, it has been shown that human GMSCs can suppress the differentiation of CD4^+^ T cells into Th1 and Th17 cells *in vitro (*52, 66). Several studies have also shown that co-culture with mouse GMSCs can robustly suppress the differentiation of mouse naïve CD4^+^ T cells into Th1, Th2 or Th17 cells under different experimental conditions (58, 59, 62, 65, 67). Meanwhile, numerous *in vivo* studies have shown that systemic administration of either human (15, 54, 56, 61, 62, 64, 65, 67, 68, 70, 72) or mouse (51, 58, 59, 63) GMSCs suppressed Th1, Th2, and/or Th17 cell differentiation and activity in various preclinical disease models. For instance, treatment with human GMSCs significantly reduced the frequency of Th2 and Th17 cells when compared to primary dermal fibroblast (PDF) treatment of lupus nephritis model in mice; meanwhile GMSCs also reduced T follicular helper (Tfh) cells in both spleen and lymph nodes (71). However, the mechanisms by which GMSCs suppress Th17 differentiation remain largely unknown. Yang R et al. reported that *Cbs^−/−^* GMSCs showed a decreased capacity to inhibit Th17 cell differentiation, while such deficient immunosuppressive effect of *Cbs^−/−^* GMSCs on Th17 cell differentiation were partially restored by NaHS supplement, suggesting that endogenous H_2_S in GMSCs may play a role in GMSC-mediated suppression of Th17 cell differentiation (58). Therefore, further studies are necessary to explore the mechanisms by which GMSCs exert their suppressive effects on Th17 cells both *in vitro* and *in vivo*.

#### Tregs

Tregs play key pivotal roles in immune homeostasis *via* counteracting the activity of various subsets of T helper cells under healthy and pathological conditions (87). It has been well documented that MSCs of different tissue origins can potently boost the differentiation and activity of Tregs both *in vitro* and *in vivo (*6). A recent study showed that human CD4^+^CD25^-^ T cells co-cultured with human GMSCs favorably differentiate into T-cell subsets displaying the regulatory phenotypes CD4^+^CD25^+^Foxp3^+^ along with an increased production of IL-10 (69). On the other hand, both human (21, 67)and mouse (58, 59) GMSCs also robustly promote the differentiation of mouse CD4^+^CD25^-^ T cells into Tregs under various *in vitro* co-cultures. Meanwhile, numerous *in vivo* studies have shown that systemic administration of either human (15, 57, 61, 62, 65, 67, 68, 72) or mouse (51, 58, 59, 63) GMSCs remarkably boost the generation of CD4^+^CD25^+^Foxp3^+^ Tregs in various preclinical disease models. Previous studies indicate that MSCs of different tissue-origins promote the generation of Tregs through their secretion of certain factors, e.g. IL-10, TGF-β1, PGE2, heme oxygenase-1 (HO1), or LIF, depending on the experimental conditions (6, 88). However, the mechanisms by which GMSCs promote the generation and functions of Tregs still remain elusive. CD39(NTPDase1) drives the hydrolysis of ATP and ADP to generate AMP, which is then hydrolyzed by CD73 (ecto‐5′‐nucleotidase) to adenosine. This metabolic pathway plays an important role in orchestrating immunomodulatory functions of various types of immune cells, particularly, Tregs (89). A previous study showed that pretreatment of human GMSCs with CD39 or CD73 inhibitors significantly abrogated GMSC-mediated increase in the frequency of FoxP3+ Treg cells and protection against the progression of mouse model of CIA (62). Most recently, Ni X et al. reported that GMSCs promote the generation and immunosuppressive function of CD39^+^Foxp3^+^ Tregs through the CD39 pathways (67). These findings suggest that CD39/CD73/adenosine pathway may play an important role in GMSC-mediated generation of Tregs.

#### B Cells

B lymphocytes play essential roles in adaptive immune responses through antibody secretion and complement activation. Previous studies have shown that MSCs are capable of suppressing B cell proliferation, differentiation, and production of antibodies (90). Meanwhile, MSCs can promote the generation of regulatory B cells (Bregs) characterized by the secretion of IL-10 and potent immunosuppressive functions (90, 91). Most recently, Dang J et al. reported that human GMSCs possess potent immunosuppressive effects on murine B cells both *in vitro* and *in vivo*. In their *in vitro* studies, they found that GMSCs could potently suppress the expression of CD69, CD25, CD80 and CD86 on B cells, suggesting that GMSCs directly suppress B cell activation [both in early activation (CD69) and late activation (CD25)] and antigen presenting ability (CD80 and CD86), whereby GMSCs suppress B cell late activation marker (CD25) more than the early activation marker (CD69) (71). Meanwhile, their results showed that GMSCs suppress B cell proliferation and the secretion of IgG and IgM partially through CD39^-^CD73 signaling pathway (71). *In vivo*, systemic administration of GMSCs remarkably reduced the expression of CD69, CD80, and CD86 on B cells and the percentage of plasma cells in lymph node and spleen as well as the secretion of total IgG and IgM autoantibodies in NZM2328 SLE mice as compared with the control. By using KLH mice model, they further showed that GMSCs significantly decreased the frequency of germinal center (GC) B cells (B220^+^ GL-7^+^ and Fas^+^) (71). These findings suggest that GMSCs are capable of suppressing B cell proliferation, differentiation, and activation both *in vitro* and *in vivo*. Further studies are necessary to explore whether GMSCs can suppress B cells and promote generation and function of Bregs under different pathological conditions.

## GMSC-Based Regenerative Therapy

**Figure 3:**
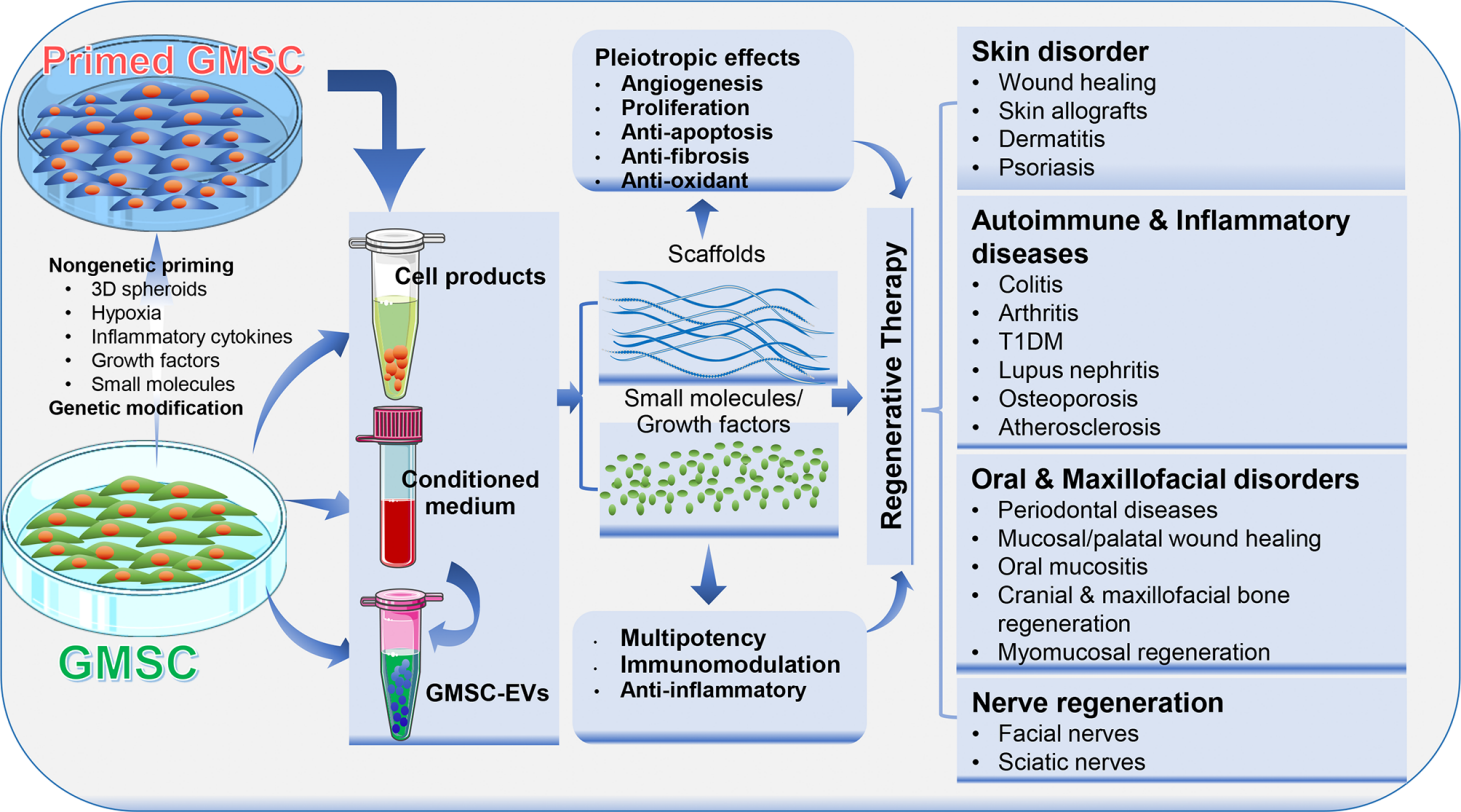
Application of GMSCs and their cell-free products in regenerative therapy. Naïve or primed GMSCs, their derivative conditioned medium or extracellular vesicles (EVs) can be administered alone or in combination with certain scaffold, growth factor or small molecules. Due to the immunomodulatory/anti-inflammatory and pleiotropic effects, GMSCs and their derivative cell-free products exert potent regenerative and therapeutic potentials in a variety of preclinical models of human disorders. Portions of this figure were made using templates from SMART SERVIER MEDICAL ART (https://smart.servier.com).

Due to their multipotent differentiation and potent immunomodulatory/anti-inflammatory capacities, MSCs and their derivative cell-free products, such as conditioned media and extracellular vesicles, have been extensively investigated as regenerative therapeutics for the treatment of a wide range of pathological conditions and diseases (4, 6). In this section, we will highlight GMSC-based regenerative therapy for a variety of disorders, such as skin disorder, autoimmune and inflammatory diseases, nerve regeneration, and oral and maxillofacial disorders (**Figure 3**).

### Skin Disorders

Up to date, a growing body of preclinical and clinical studies have demonstrated the potential application of MSCs and their cell-free products in the treatment of various skin disorders (92). A panel of studies have also demonstrated the therapeutic potentials of GMSCs and their derivative cell-free conditioned medium or EVs in skin disorders, particularly full-thickness skin wound and dermatitis (**Table 3**).

**Table 3:** GMSC-based regenerative therapy of skin disorders.

Cell products	Other factors	Dose	Route	Model	Refs
GMSC		2×10^6^/mice	i.v.	Mice, full-thickness excisional skin wound	Zhang et al. (52)
GMSC	Hypoxia-primed	2×10^6^/mice	i.v.	Mice, skin wound	Jiang et al. (60)
GMSC	3D-printed medical-grade polycaprolactone (mPCL) fibrous network dressings	1.0 × 10^5^cells /dressing.	Local	Rat, full-thickness splinted excisional skin wound	Shafiee et al. (93)
GMSC	Nanofibrous Guar gum/PVA based scaffold matrix, extracts of medicinal plants of wound healing repute	5.0 × 10^4^ cells /construct	Local	Rat, full-thickness splinted excisional skin wound	Kalachaveedu et al. (94)
GMSC	IL-1β-primed	7.5×10^5^/mice	Local	NOD/SCID mice; excisional skin wound /epidermal substitute engraftment	Magne et al. (95)
GMSC-EV	Chitosan/Silk Hydrogel Sponge	150 μg/wound	Local	Rat diabetic skin defects	Shi et al. (96)
GMSC-EV		40µg/wound	Local	Mice, full-thickness excisional skin wound	Kou et al. (97)
GMSC-EV	TNFα-primed	200µg/wound	Local	Mice, full-thickness excisional skin wound	Nakao et al. (98)
GMSC		1 × 10^6^ cells /mice	i.v.	Mice skin allografts	Tang et al. (21)
GF	Gingival fibroblasts		Local	Mice, cutaneous radiation syndrome	Linard et al. (53)
GMSC		2.0 × 10^6^/mice	i.v.	Mice, skin contact hypersensitivity (CHS)	Su et al. (56)
GMSC		2.0 × 10^6^/mice	i. v. /local	Mice, skin contact hypersensitivity (CHS)	Li et al. (61)
GMSC	Human case study	3 × 10^6^/Kg /infusion		Plaque psoriasis	Wang et al. (99)

#### Skin Wound

Our group first showed that systemically administered human GMSCs into immunocompetent mice can migrate to the local skin wound beds, accelerate skin wound closure, promote re-epithelialization, angiogenesis, and suppress local inflammatory cell responses (52). Afterwards, another study showed that human GMSCs pre-conditioned by hypoxia exhibited enhanced reparative effects on full-thickness skin wounds in mice (60). Most recently, Shafiee A et al. reported that local application of 3D-printed medical-grade polycaprolactone (mPCL) dressings remarkably reduced wound contracture and facilitated skin tissue granulation and re-epithelialization, whereas combining 3D-printed biomimetic wound dressings and GMSCs enhances physiological wound closure with reduced scar tissue formation in a splinted full-thickness excisional wound in a rat model (93). Interestingly, local implantation of an herb drug-enriched nanofibrous scaffolds seeded with human GMSCs also improved wound healing and tissue restoration with minimal scarring in a rat model of splinted full-thickness excisional wound (94). Using an excisional skin wound/human epidermal substitute engraftment model in NOD/SCID mice, Magne B et al. have recently shown that IL-1β-primed GMSCs promoted cell migration, dermal-epidermal junction formation, and inflammation reduction *in vitro*, and improved epidermal substitute engraftment and skin wound healing *in vivo (*95). Additionally, several studies indicate that local application of GMSC-EVs also display potent beneficial effects to promote full-thickness skin wound healing in mice (97, 98), and even in diabetic skin defects in rats (96).

#### Skin Allografts

An early study showed that systemic administration of human GMSCs exhibited stronger beneficial effects than their BMSC counterparts in terms of prolonging the skin allograft survival and delayed the allograft rejection, which may be attributed to increased function of Tregs *in vivo (*21).

#### Contact Hypersensitivity

Using a murine model of skin contact hypersensitivity (CHS), we found that systemic infusion of GMSCs before the sensitization and challenge phase dramatically attenuated CHS as evidenced by reduced infiltration of dendritic cells (DCs), CD8^+^ T cells, Th17, and mast cells (MCs), a decreased level of several inflammatory cytokines, and simultaneously, an increase in the infiltration of regulatory T cells and expression of IL-10 at the regional lymph nodes and the allergic contact areas (56). Meanwhile, another study further demonstrated the attenuative effects of GMSCs on skin CHS in mice, wherein it was reported that local injection of GMSCs exhibited more potent suppressive effects on the manifestation of CHS, especially during the late phase of CHS, than intravenous injection of GMSCs (61). Both studies indicate that COX2/PGE_2_ signaling plays an important role in GMSC-mediated attenuation of skin CHS in mice (56, 61).

#### Psoriasis

Psoriasis is a chronic, relapsing, and systemic inflammatory disease, which affects 2–3% of the population (100). Due to the lack of effective therapeutics for this disease, numerous preclinical and clinical studies have endeavored to explore the therapeutic potential of MSCs of different tissue-origins for the treatment of psoriasis (100). Interestingly, Wang SG et al. have recently reported a case of a 19-year-old man with a 5-year history of severe plaque psoriasis refractory to multiple topical and systemic therapies, who then received two successive weekly administrations of allogeneic human GMSCs (3 × 10^6^/Kg/infusion). Clinically, complete regression was achieved after 5 infusions with no adverse reactions. The patient has been followed for three years and has remained disease free (99).

### Autoimmune and Inflammatory Diseases

Due to their potent immunomodulatory effects on various subtypes of immune cells, different sources of MSCs have shown great promises in regenerative therapy of a wide range of autoimmune and inflammatory diseases (6). Similarly, GMSCs have also been shown to possess therapeutic potentials in several preclinical models of autoimmune and inflammatory diseases (**Table 4**).

**Table 4:** GMSC-based regenerative therapy in autoimmune and inflammatory disorders.

Cell products	Dose	*Route*	*Model*	Refs
GMSC	2×10^6^/mice	i.v.	Mice, colitis (DSS)	Zhang et al. (15)
GMSC	n/a	i.v.	Mice, colitis (DSS)	Lu et al. (57)
GMSC	2 × 10^5^/mice	i.v.	Mice, colitis (DSS)	Xu et al. (51)
GMSC	2 × 10^5^/mice	i.v.	Mice, colitis (DSS)	Yang et al. (58)
GMSC	1 × 10^6^/mice	i.v.	Mice, colitis (DSS)	Yu et al. (59)
GMSC	2 × 10^6^/mice	i.v.	Mice, CIA arthritis	Chen et al. (62)
GMSC	1 × 10^6^/mice	i.v.	Mice, CIA arthritis	Gu et al. (63)
GMSC	2×10^6^/mice	i.v.	Mice, CIA arthritis	Luo et al. (64)
GMSC	2 × 10^6^/mice	i.v.	Mice, CIA arthritis	Wu et al. (65)
GMSC	5 × 10^6^/mice	i.v.	Mice, GvHD	Huang et al. (66)
GMSC	2 × 10^6^/mice	i.v.	Mice, GvHD	Ni et al. (67)
GMSC	1 × 10^6^/mice 1dose/week	i. p. 5 doses	Mice, T1DM	Zhang et al. (68)
GMSC	2 × 10^6^/mice	i.v.	Mice, SLE nephritis	Dang et al. (71)
GMSC	2 × 10^6^/mice	i.v.	Mice, osteoporosis	Wu et al. (72)
GMSC	2 × 10^6^/mice	i.v.	AopE^-/-^ mice, Atherosclerosis	Zhang et al. (54)
GMSC	2 × 10^6^/mice	i.v.	Mice, Bone marrow failure/Aplastic anemia	Zhao et al. (70)

#### Colitis

Our group is the first to explore the therapeutic effect of human GMSCs on dextran sulfate sodium (DSS)-induced acute colitis in mice and found that systemic infusion of GMSCs ameliorated both clinical and histopathological severity of the colonic inflammation, restored the injured gastrointestinal mucosal tissues, and improved the overall disease score (15).** **Most recently, Lu Y et al. also reported that systemic infusion of human GMSCs significantly prolonged survival and attenuated disease manifestations, whereby IL-10/IL-10R signaling may play a critical role in GMSC-mediated therapeutic effects on colitis (57). In addition, several lines of evidence have demonstrated that systemic infusion of mice GMSCs also markedly attenuate the severity of DSS-induced acute colitis in mice, whereby the expression of FasL play an important role in GMSC-mediated therapeutic effects on colitis in mice (51, 58, 59). Interestingly, Xu X et al. reported that neural crest-derived GMSCs with increased expression of FasL show superior therapeutic effects on DSS-induced colitis in comparison with the mesenchymal-derived GMSC counterparts (51). On the other hand, Yang R et al. found that H_2_S-deficient GMSCs exhibit attenuated therapeutic effects on colitis *in vivo*, which could be restored by treatment with the H_2_S donor, NaHS (58). Taken together, these studies have demonstrated the therapeutic potential of GMSCs in the treatment of colitis.

#### Arthritis

In an established collagen‐induced arthritis (CIA) model in DBA/1J mice, Chen M et al. showed that systemic infusion of human GMSCs markedly ameliorated the severity of arthritis, reduced the histopathological scores and the production of inflammatory cytokines, while pretreatment of GMSCs with a CD39 or CD73 inhibitor significantly attenuated the protective effect of GMSCs on CIA (62). Gu Y et al. reported that systemic infusion of mouse GMSCs also significantly attenuated the severity of experimental CIA in DBA/1J mice, while FasL^–/–^ GMSCs showed no therapeutic effects, suggesting that FasL signal plays a critical role in GMSC-mediated therapeutic effects on CIA (63). Most recently, a similar study showed that systemic infusion of human GMSCs into mice remarkably attenuated the severity of CIA, the pathological score, the frequency of osteoclasts, particularly bone erosion *in vivo*, while blockade of CD39/CD73 or adenosine receptors significantly abolished the therapeutic effect of GMSC on bone erosion during CIA *in vivo (*64). Most recently, Wu W et al. showed that B7-H1(PD-L1) plays an important role in the immunosuppressive and therapeutic effects of human GMSCs in CIA, which is dependent on STAT3 signaling pathway (65). Taken together, these studies have demonstrated the therapeutic potential of GMSCs in the treatment of arthritis.

#### GvHD

Using a xenogenic GvHD model through the transfer of human CD25-depleted PBMC to NOD/SCID mouse manifested with weight loss, elevated human pro-inflammatory cytokines, anti-human antibodies, and death of the host animal at around 2 weeks, Huang F et al. reported that co-transfer of GMSCs significantly prolonged mouse survival, suppressed the expansion of human T cells in the mouse, and prevented weight loss. Meanwhile, co-transfer of GMSCs markedly ameliorated the pathological changes and inflammation degrees, such as necrosis and lymphocyte infiltration in liver, lung, and intestine (66). Most recently, Ni X et al. reported that systemic infusion of human GMSCs significantly attenuated the lethal acute GvHD in two mouse models, C57BL/6-to-BALB/c and C57BL/6-to-B6D2F1, respectively (67). Meanwhile, their results indicate that GMSCs exhibit greater therapeutic potentials against acute GvHD than BMSC and ADSC, whereby CD39/adenosine signaling plays an important role (67).

#### T1DM

Using a murine T1DM model induced by multiple injections of low-dose streptozotocin (STZ), Zhang W et al. reported that adoptive transfer of human GMSCs led to a robust control of blood glucose levels, delayed diabetes onset, ameliorated pathologic scores in pancreas, down-regulated production of IL-17 and IFN-γ in CD4^+^ and CD8^+^ T cells in spleens, pancreatic lymph nodes (pLN) and other lymph nodes as well as up-regulated levels of periphery CD4^+^ Treg (68). Mechanistically, their results indicate that GMSCs could migrate to pancreas and local lymph nodes to regulate effector T cells through CD39/CD73 signaling pathway (68).

#### Lupus Nephritis

Using a spontaneous lupus nephritis model in mice, Dang J et al. have recently reported that adoptively transferred human GMSCs could home to and be retained in the kidney, and significantly ameliorated the spontaneous lupus nephritis. Meanwhile, systemic infusion of GMSCs remarkably reduced the production of autoantibodies, development of proteinuria, frequency of plasma cells, and histopathological scores of lupus nephritis by directly suppressing B cells activation, proliferation, and differentiation through targeting of CD39 (–) CD73 signaling pathway (71).

#### Osteoporosis

Using an ovariectomy (OVX)-induced osteopenic mouse model, Wu W et al. has recently reported that systemic administration of human GMSCs significantly attenuated OVX-induced osteoporosis as evidenced by increased trabecular bone densities and frequency of osteoblasts but a decreased frequency of osteoclasts. Meanwhile, their results indicate that a unique population of CD39^+^ GMSC play a critical role in promoting bone formation, whereby CD39 produced from GMSC exerted its osteogenic capacity through the Wnt/β-catenin pathway (72).

#### Atherosclerosis

With the use of an atherosclerosis model in apolipoprotein E knock out (ApoE^−/−^) mice, Zhang X et al. have reported that systemic infusion of human GMSC to ApoE^-/-^ mice significantly reduced the plaque size and lipid deposition in the wall of blood vessels, the frequency of inflammatory monocytes/macrophages, and macrophage foam cell formation. These results suggest that GMSCs exert their therapeutic effects on atherosclerosis *via* inflammatory monocytes/macrophages (54).

#### Bone Marrow Failure

Human aplastic anemia (AA), a rare autoimmune disease, is manifested by severe pancytopenia and bone marrow failure (BMF). Using a mouse model of T cell-mediated BMF, Zhao J et al. have recently shown that systemically infused human GMSCs could home into inflammatory location in bone marrow (BM), remarkably improved mice survival, and ameliorated the histological damage score of BM (70). Mechanistically, their results indicate that GMSCs mitigate T cell-mediated BMF through regulating the balance of Th1, Th17, and Tregs (70).

### Oral and Craniofacial Disorders

In addition to their potential application in regenerative therapy for a variety of skin and systemic disorders, GMSCs and their derivative cell-free products alone or in combination with different scaffolds have also been widely explored in the treatment of various preclinical animal models of oral and craniofacial disorders (**Table 5**) (121).

**Table 5:** GMSC-based regenerative therapy in oral & craniofacial disorders.

Cell Type	Scaffolds/other factors	Dose	Model		Refs
GMSC-CM	Collagen scaffolds (Bio-Gide) Ultra-15 10 kD, 100-fold CM	2 mm × 3 mm	Periodontal defects	Rats	Qiu et al. (101)
GMSC-Exo	Exosomes, TNFa-preconditioned	20µg/mice	Periodontitis bone loss	Mice	Nakao et al. (98)
GMSC	i.v. injection	1×10^6^/mice	Periodontitis bone loss	(ApoE^-/-^) mice	Liu et al. (102)
GMSC	i.v. injection	1×10^6^/mice	Periodontitis	Mice	Sun et al. (103)
GMSC	i.v. injection		Class III furcation defects	Dog	Yu et al. (104)
GMSC	IL-1Ra-hyaluronic acid synthetic extracellular matrix (HA-sECM)	250 *μ*l GMSCs/HA‐sECM (5×10^6^ cells)	Periodontal defects	minipig	Fawzy El-Sayed et al. (105)
GMSC	β-TCP scaffold and covered by a collagen membrane	2 × 10^5^ to 8 × 10^6^ cells/cm^3^	Periodontal defects	Human	Abdal-Wahab et al. (106)
GMSC	alginate-based adhesive, photocrosslinkable hydrogel (AdhHG)	4 × 10^6^/construct	Peri-implantitis model	Rat	Hasani-Sadrabadi et al. (107)
GMSC	IV injection	1 × 10^6^/mice	Mandibular bone	Mice	Xu et al. (108)
GMSC	Type I collagen		Mandibular defect Calvarial defect	Rat	Wang et al. (25)
GMSCs	Hydrogel scaffold PuraMatrix™ (PM)/BMP2	1 × 10^6^/rat	Maxillary alveolar defect	Nude rats	Kandalam et al. (109)
GMSCs	Bio-Oss^®^/SB431542	2 × 10^6^/pig	Maxillary bone defects	minipigs	Shi et al. (110)
GMSC-EV	Poly(lactide) (3D-PLA) 3D printing	2 × 10^3^/scaffold 0.5µg EV/µl	Calvarial defect	Rat	Pizzicannella et al. (111)
GMSC-EV	(3D) engineered scaffolds (PLA)	2 × 10^6^/scaffold	Calvarial defect	Rat	Diomede et al. (112)
GMSC-CM	a poly-(lactide) (3D-PLA) scaffold enriched with GMSCs and GMSCs derived CM	2 × 10^6^/scaffold EVs?	Calvarial defect	Rat	Diomede et al. (113)
GMSC-EVs		40µg/mice	Palatal wound	Mice	Kou et al. (97)
Fetal GMSCs		2 × 10^6^ cells/rat	gingival defects	Rat	Li et al. (114)
GMSCs	3D spheroids 2D GMSC i.v. injection	1× 10^6^/mice 2× 10^6^/mice	Chemotherapy-induced oral mucositis	Mice	Zhang et al. (115)
GMSC	SIS-ECM, 5 × 4 mm	3.5 × 10^5^ cells/cm^2^	Tongue defects	Rat	Xu et al. (116)
GMSC/EVs	SIS-ECM, 5 × 4 mm	3.5 × 10^5^ cells/cm^2^	Tongue defects	Rat	Zhang et al. (117)
GMSC	Fibrin glue	1 × 10^5^ cells/rat	Partially dissected submandibular salivary glands	Rat	Abd El-Latif et al. (118)
GMSC-NC	AxoGuard nerve conduits	0.5×10^6^ cells/rat	Facial nerve	Rat	Zhang et al. (119)
GMSC	3D spheroid, 3D bioprinting		Facial nerve	Rat	Zhang et al. (120)

#### Periodontal Diseases

With the use of an established periodontitis model in mice, Sun W et al. have recently shown that systemically administered human GMSCs could home to periodontal inflammatory sites and promote periodontal tissue regeneration as evidenced by significantly increased alveolar bone heights as compared with the control groups (103). In a severe periodontitis model developed in apolipoprotein E-deficient (ApoE^-/-^) mice with a hyperlipidemia, Liu X et al. showed that systemic infusion of human GMSCs not only attenuated the hyperlipidemia and systemic inflammatory responses but also promoted periodontal tissue regeneration as evidenced by increased alveolar bone height and new bone formation compared to the control groups (102). In the class III furcation defect model in beagle dogs, systemically administered GMSCs were able to home to the injury site and differentiate into osteoblasts, cementoblasts, and PDL fibroblasts *in vivo*, and remarkably enhanced the regeneration of the damaged periodontal tissue, including the alveolar bone, cementum and functional periodontal ligament (PDL) (104). In addition, Fawzy El-Sayed KM et al. have demonstrated that implantation of autologous GMSCs in conjunction with IL‐1ra‐loaded/A‐sECM in the premolar/molar area significantly facilitated periodontal regeneration in a periodontal defect model in miniature pigs (105). Most interestingly, Abdal-Wahab M et al. have recently performed a randomized clinical trial study to explore the potential application of gingival fibroblasts (GF) and their associated mesenchymal stem cells (GMSC) in the treatment of intrabony periodontal defects (106). The results indicate that transplantation of autologous GMSC-loaded β‐calcium triphosphate (β‐TCP) into the intrabony periodontal defects followed by collagen membrane defect coverage significantly reduced the vertical pocket depth (VPD) and clinical attachment loss (CAL) and enhanced the radiographic bone gain as compared with the β‐TCP control group 6 months post-surgery (106). In addition, local application of GMSC-derived conditioned medium or exosomes also exhibited potent therapeutic effects on periodontitis in rats (101) and mice (98), respectively. Altogether, these studies have demonstrated the promising potentials of GMSC-based regenerative therapy for treating periodontal diseases.

#### Peri-Implantitis

In a well-established rat peri-implantitis model, Hasani-Sadrabadi MM et al. recently reported that local injection of the adhesive hydrogel (AdhHG) encapsulating human GMSCs into the bony defect sites around the implants rescued the implants in all the experimental rats, significantly promoted bone formation and reduced the local inflammatory responses at the peri-implantitis sites. These results have demonstrated the potential application of GMSCs in regenerative therapy of peri-implantitis (107).

#### Maxillofacial Bone Defects

Several preclinical studies have shown the therapeutic and regenerative potential of GMSCs in mandibular bone defects. A previous study showed that systemically infused GMSCs can home to injury site and significantly promote new bone formation at the mandibular defects (108). In a rat mandibular defect model, locally transplanted human GMSCs mixed with type I collagen gel can differentiate into osteocytes *in vivo* and contribute to new bone formation at the bone defects, thus facilitating mandibular wound healing (25). In a recent study, hydrogel scaffold PuraMatrix™ (PM)/BMP2, PM/dGMSCs or the combination of three (PM/dGMSCs/BMP2) were filled in critical-sized maxillary alveolar defects in nude rats, and then, the bone regeneration was evaluated at 4 and 8 weeks post-surgery (109). The results indicate that PM/dGMSCs/BMP2 group exhibit significantly enhanced bone regeneration compared to groups with the transplantation of individual material/cells alone, suggesting the bone regenerative potential of pre-differentiated GMSCs (109). Most recently, Shi A et al. reported that local transplantation of autologous pig GMSCs loaded with Bio-Oss^®^, particularly pGMSCs treated by SB431542, a specific TGF-β signaling inhibitor, remarkably facilitated repair/regeneration of severe maxillofacial bone defects in minipigs (110).

#### Calvarial Bone Defects

A previous study has shown that locally transplanted human GMSCs mixed with type I collagen gel can differentiate into osteocytes *in vivo* and promote new bone formation at the calvarial bone defects in rats (25). In a critical-sized rat calvarial defect model, local transplantation of 3D-printed poly(lactide) (3D-PLA) loaded with GMSCs or GMSC-EVs promote bone regeneration along with an enhanced vascularization in calvaria defects (111). In two earlier studies, Diomede F et al. also reported that 3D-engineered scaffolds (PLA) loaded with either GMSCs or GMSC-EVs, or GMSC-derived conditioned medium (CM) significantly enhanced bone regeneration in rat calvarial defects (112, 113). Altogether, these studies have demonstrated the therapeutic and regenerative potentials of GMSCs in regenerative therapy of craniomaxillofacial bone defects.

#### Palatal/Gingival Defects

It has been shown that endogenous GMSCs play an important role in tissue homeostasis and wound healing (17, 122). In a gingival defect model in rats, local transplantation of human fetal GMSCs around the defect markedly facilitated gingival wound closure and re-epithelialization at one week following transplantation, wherein the morphology and color of local gingival tissue was similar to normal gingival tissue and gingival height was the same as the normal control group three weeks after transplantation (114). In addition, local injection of extracellular vesicles derived from mice GMSCs also significantly promoted palatal defect healing process in mice whereby IL-1Ra may play an important role (97).

#### Oral Mucositis

Our previous study showed that systemic administration of human GMSCs or 3D-GMSC spheroids obviously mitigated chemotherapy-induced oral mucositis in mice. This was evidenced by the reverse of body weight loss and acceleration of the regeneration of disrupted epithelial lining of the mucositic tongues, thus suggesting the potential application of GMSCs in the regenerative therapy of chemotherapy-related mucositis (115).

#### Tongue Defect and Muscle Regeneration

A previous study by Ansari et al. showed that human GMSCs encapsulated in an injectable 3D RGD-coupled alginate scaffold with multiple growth factors displayed potent myogenic differentiation potentials as evidenced by muscle cell-like morphology with high expression levels of muscle regeneration-related genes both *in vitro* and *in vivo* when encapsulated GMSCs were subcutaneously transplanted into immunocompromised mice. Interestingly, their results indicated that GMSCs exhibited significantly greater capacity for myogenic regeneration compared to human bone marrow MSCs, suggesting that GMSCs could be a promising candidate source of cells for muscle tissue engineering  (123). Most recently, our group established a critical-sized myomucosal tongue defect model in rats to test the therapeutic effects of GMSCs on myomucosal regeneration (116, 117). We found that local transplantation of porcine small intestine submucosal-extracellular membrane (SIS-ECM) loaded with human GMSCs remarkably facilitated the tongue defect healing as evidenced by accelerated wound closure, re-epithelialization, regeneration of taste bud and the muscular layer as compared to transplantation of the SIS-ECM alone or nontreated defect controls (116, 117). Meanwhile, we showed that transplantation of GMSC/SIS-ECM constructs markedly upregulated the expression of several myogenic transcriptional factors (e.g. MyoD, PAX7, Myf5), growth factors (e.g. BDNF and Shh), neurofilament (a neuronal marker), and concomitantly, reduced fibrosis at the wound healing site (116, 117). These findings suggest that transplantation of GMSC/SIS-ECM constructs might be a novel approach for regenerative tongue reconstruction and rehabilitation post-surgery.

#### Regeneration of Submandibular Salivary Glands

In a rat model of partially dissected submandibular salivary glands, a previous study showed that local application of human GMSCs mixed with fibrin glue markedly promoted the regeneration of ductal, acinar, and myoepithelial cells of salivary glands as compared to rats that received no treatment or treated with fibrin glue alone (118).

#### Facial Nerve Regeneration

Recently, our studies have shown that GMSCs or GMSC-derived neural crest cells have the potential to facilitate facial nerve regeneration (119, 120) as described in detail in the following section of nerve regeneration.

### Nerve Regeneration

Due to their neural crest origin (17, 51), GMSCs are inclined to differentiate into functional neuronal and glial types of cells (34) and have the propensity to be reprogrammed into neural progenitor or neural crest-like stem cells (119, 124). Most recently, several lines of evidence have demonstrated the nerve regenerative potentials of GMSCs and their derivative EV products under different experimental conditions (**Table 6**).

**Table 6:** GMSC-based regenerative therapy for nerve regeneration.

Cell Type	Scaffolds/other factors	Dose	Model		Refs
GMSC-NC	AxoGuard nerve conduits	0.5×10^6^ cells/rat	Facial nerve defect	Rat	Zhang et al. (119)
GMSC	3D spheroid, 3D bioprinting		Facial nerve defect	Rat	Zhang et al. (120)
GMSC-NPC	GelFoam	2×10^5^ cells/rat	Sciatic nerve crush-injury	Rat	Zhang et al. (124)
GMSC-EVs	GelFoam	40µg/mice	Sciatic nerve crush-injury	Mice	Mao et al. (125)
GMSC-EVs	Chitin conduits	10 *μ*g/rat	Sciatic nerve segmental defect, 10 mm gap	Rat	Rao et al. (126)
GMSC	Liposome enriched with moringin (MOR) treatment	1×10^6^ cells/mice, i.v.	Spinal cord injury (SCI)	Mice	Mammana et al. (127)
GMSC	Caffeic acid‐based bioconjugated hydrogel (CBGH)	n/a	SCI (hemitransection model)	Rat	Subbarayan et al. (128)

#### Facial Nerve Regeneration

In our recent study, we have shown that human GMSCs can be reproducibly induced or reprogrammed into neural crest stem-like cells (NCSC) under defined culture conditions without genetic introduction of specific transcriptional factors into the cells (119). Implantation of collagen nerve conduits filled with GMSC-derived NCSCs mixed in Matrigel in a facial nerve defect model in rats significantly enhanced the functional recovery and axonal regeneration of the injured nerve as compared to transplantation of GMSC-laden nerve conduits (119). Interestingly, using a state-of-the-art 3D bio-printer system, we printed scaffold-free and implantable nerve grafts from GMSC 3D-spheroids enriched with NCSC-like properties. Implantation of 3D-bioprinted GMSC-laden nerve grafts to the facial nerve defect in rats remarkably facilitates functional recovery and axonal regeneration of injured facial nerves as compared to transplantation of the empty silicon nerve conduits (120).

#### Sciatic Nerve Regeneration

Recently, we also showed that human GMSCs can be consistently induced into neural progenitor-like cells (NPC) under defined culture conditions without genetic introduction of specific transcriptional factors into the cells (124). Using a sciatic crush-injury model in rats, we found that GMSCs transplanted to the injury site could differentiate into neuronal cells, while GMSC-derived NPCs could differentiate into both neuronal and Schwann-like cells *in vivo*. Meanwhile, we showed that transplantation of GMSC-derived NPCs displayed superior therapeutic effects, as compared to transplantation of parental GMSCs, on axonal regeneration at both the injury and the distal sites of sciatic nerves (124). In addition, our recent study indicated that local delivery of GMSC-EVs significantly facilitates axonal regeneration and functional recovery of crush-injured sciatic nerves in mice (125). Mechanistically, our findings suggest that GMSCs, GMSC-derived NPCs, or GMSC-EVs promote peripheral nerve regeneration possibly by promoting the reprogramming of Schwann cells into a repair phenotype characterized by increased proliferation and expression of a key transcriptional factor, c-JUN (124, 125). Most recently, Rao F et al. also reported that GMSC-derived exosomes could promote Schwann cell proliferation and DRG axon growth *in vitro (*126). Using a sciatic nerve segmental defect model in rats, they showed that transplantation of GMSC exosome-loaded chitin conduits significantly enhanced the recovery of muscle function, nerve conduction, and motor function, and robustly increased the number and diameter of nerve fibers and the thickness of myelin sheath (126).

#### Spinal Cord Injury

In spite of the potent regenerative potentials of GMSCs in peripheral nerve injuries, they have been shown to promote repair/regeneration of spinal cord injury (SCI). Mammana S et al. recently reported that intravenous injection of GMSCs pretreated with liposome enriched with moringin (MOR) into mice with SCI significantly promoted SCI repair possibly through suppressing the local inflammatory responses and apoptotic pathways (127). In a hemitransection spinal cord injury (SCI) model in rats, transplantation of human gingival derived neuronal stem cells encapsulated in the injectable caffeic acid bioconjugated hydrogel (CBGH) significantly reduced the local inflammatory responses, promoted the engraftment and repopulation of neural cells the injured spinal tissue, and facilitated the new synaptic vesicle formation and functional improvements (128). These findings suggest potential application of GMSCs in regenerative therapy of spinal cord injuries.

### Bone and Cartilage Regeneration

Tissue engineering (TE) involves the combined use of various types of seed cells, scaffolds, and growth factors (129). A growing body of evidence has demonstrated the potential application of GMSCs in tissue engineering of different types of tissues, including cartilage and bone tissues. When cultured on chitosan membranes, gingival stromal cells formed spheroids and showed enhanced chondrogenic potentials (49). A previous study showed that the sustained release of TGF-β1 from RGD-modified alginate microspheres significantly promoted *in vitro* chondrogenic differentiation potential and *in vivo* ectopic cartilage tissue formation capacity of PDLSCs and GMSCs (130). These studies suggest that combination with certain types of biomaterials can guide and improve the chondrogenic differentiation and cartilage regenerative potentials of GMSCs.

Several studies have also demonstrated the enhanced osteogenic differentiation potentials of GMSCs in combination with different types of biomaterials. For instance, Ansari S et al. reported that GMSCs encapsulated in alginate hydrogel without gelatin methacryloyl (GelMA) underwent osteogenic differentiation without the aid of additional growth factors, suggesting the possibility to control the fate of encapsulated MSCs within hydrogels by tuning the mechanical properties of the matrix (131). Most recently, a study has shown that local application of GMSCs can promote long bone regeneration (132). Using a critical-sized tibiae defect model in rabbit, Al-Qadhi G et al. showed that local transplantation NanoBone scaffolds loaded with GMSCs or BMSCs significantly enhanced the new bone formation as compared to the unloaded scaffolds, whereby GMSCs and BMSCs exhibited similar degree of bone regenerative potentials in bone defects in rabbits’ tibiae, suggesting that GMSCs could be a comparable alternative source to BMSCs for bone regeneration (132).

## Potential Application of GMSC-EVs in Tissue Engineering and Regenerative Medicine

Extracellular vesicles (EVs), including ectosomes and exosomes, play important roles in intercellular communication due to their horizontal transfer of cargoes containing bioactive molecules such as lipids, nucleic acids, proteins, and metabolites, etc. (133, 134). Ectosomes, with size ranging from ~50nm to 1000nm in diameter, include microvesicles, microparticles, and large vesicles, which are generated by the direct outward budding of the plasma membrane (133). EVs secreted by various types of cells, including MSCs (MSC-EVs), possess complex effects on various biological processes, such as immune responses, cellular survival/apoptosis and proliferation, differentiation, migration, and angiogenesis (133, 135, 136). A growing body of evidence has shown that MSC-EVs exhibit potent therapeutic effects on a variety of preclinical models of oral and maxillofacial disorders (137), suggesting that application of MSC-EVs could be a promising cell-free approach for regenerative therapy due to their relatively stable properties and reduced safety risks compared to their producer cells (135, 136). In recent years, it has been shown that application of GMSC-EVs alone or in combination with different types of scaffolds possess potent therapeutic potentials in several preclinical disorder models (**Table 7**), including full-thickness skin wound (97, 98), especially the rat diabetic skin defects (96), palatal wound (97), periodontitis (98, 101), and rat cavarial defects (111–113). Our recent study showed that local implantation of SIS-ECM constructs loaded with GMSC-EVs significantly promoted myomucosal and taste bud regeneration in a critical-sized tongue defect model in rats (117). In addition, local application of GMSC-EVs have been shown to promote functional recovery and axonal regeneration in a sciatic nerve crush-injury model in mice (125) and a sciatic nerve segmental defect model in rats (126), respectively.

**Table 7:** Potential application of GMSC-EVs and conditioned medium in regenerative therapy.

Cell Type	Scaffolds/other factors	Dose	Models		Refs
GMSC-EV	Chitosan/Silk Hydrogel Sponge	150 μg/wound	Diabetic skin defects	Rat	Shi et al. (96)
GMSC-EV		40µg/wound	Excisional skin wound Palatal defects	Mice	Kou et al. (97)
GMSC-Exo	Exosomes, TNFa-preconditioned	20µg/mice	Excisional skin wound Periodontitis	Mice	Nakao et al. (98)
GMSC-CM	Collagen scaffolds (Bio-Gide), Ultra-15 10 kD, 100-fold CM	2 mm × 3 mm	Periodontal defects	Rat	Qiu et al. (101)
GMSC-EV	Poly(lactide) (3D-PLA), 3D printing	2 × 10^3^/scaffold 0.5µg EV/µl	Cavarial defect	Rat	Pizzicannella et al. (111)
GMSC-EV	(3D) engineered scaffolds (PLA)	2 × 10^6^/scaffold EV dose?	Cavarial defect	Rat	Diomede et al. (112)
GMSC-CM	a poly-(lactide) (3D-PLA) scaffold enriched with GMSCs and GMSCs derived CM	2 × 10^6^/scaffold EV dose?	Cavarial defect	Rat	Diomede et al. (113)
GMSC/EVs	SIS-ECM, 5 × 4 mm	3.5 × 10^5^ cells/cm^2^	Tongue defects	Rat	Zhang et al. (117)
GMSC-EVs	GelFoam	40µg/mice	Sciatic nerve crush-injury	Mice	Mao et al. (125)
GMSC-EVs	Chitin conduits	10 *μ*g/rat	Sciatic nerve segmental defect, 10 mm gap	Rat	Rao et al. (126)
GMSC-EVs	i.v. injection	100µg/mice x2 1 × 10^6^ cells/mice	Aged mice	Mice	Shi et al. (138)

Most recently, we have shown that GMSC-EVs can significantly inhibit oxidative stress-induced cellular senescence in both endothelial cells and skin fibroblasts, while systemic infusion of GMSC-EVs into aged mice robustly attenuated aging-associated increase in the expression of senescence-related genes in skin and heart tissues of aged mice (138). These findings suggest that GMSC-EVs have anti-aging potentials and can be developed as a cell-free therapeutics for treatment of aging-related skin and vascular disorders (138).

## Effects of Infection/Inflammation on GMSCs

According to epidemiology, a majority of adults are affected by mild to moderate periodontal diseases while about 5% to 20% of any population suffer from severe periodontitis, the main cause of tooth loss (139). GMSCs are constantly exposed to a special environment in the oral cavity that is characterized by the colonization of a complex microbial flora, play an important role in tissue homeostasis and wound healing, and are prone to be affected by acute and chronic inflammation during periodontal diseases (122, 140). Therefore, it is of great importance to explore whether an inflammatory environment has any negative effects on the property and function of GMSCs. However, currently available reports about the effects of bacteria and inflammation on GMSCs are controversial (**Table 8**). Several lines of evidence indicate that GMSCs derived from inflamed gingival tissues exhibit similar phenotypes and minimal functional changes as compared with those derived from healthy gingival tissues (140–142), while under certain situations inflamed GMSCs even showed increased proliferative activity (142) and osteogenic potentials (140). On the contrary, some studies have shown that GMSCs derived from inflamed gingival tissues exhibited phenotypic alterations and significant impairment in their functions as compared to the healthy GMSCs. For instance, Yu T et al. reported that mice GMSCs derived from inflamed gingival tissues showed decreased expression of FasL, impaired immunomodulatory effects on T cells *in vitro*, and attenuated therapeutic effects on murine colitis *in vivo (*59). Jauregui C et al. showed that inflamed GMSCs have a decreased colony-forming unit (CFU) efficiency, decreased osteogenic potentials and increased adipogenic propensity as compared with their healthy counterparts (143). In addition, an early study also showed that inflamed GMSCs had increased proliferative activity but reduced osteogenic & adipogenic potentials, and exhibited a pro-fibrotic phenotype characterized by an increased expression of inflammatory factors (144).

**Table 8:** Effects of bacteria/inflammation on the property and function of GMSCs.

Inflammatory Factors	Effects	Refs
iGMSC vs normal GMSC	Similar phenotypes Increased osteogenic potentials	Tomasello et al. (140)
	Similar phenotypes Decreased expression of Nanog, Oct3/4 and Sox-2	Soancă et al. (141)
	Similar phenotypes Increased proliferation No difference in cell migration	Al Bahrawy et al. (142)
	Decreased expression of FasL and impaired immunomodulation on T cells *in vitro* Impaired therapeutic effects on mice colitis	Yu et al. (59)
	Reduced CFU & osteogenic potential Increased adipogenic potential	Jauregui et al. (143)
	Reduced osteogenic & adipogenic potentials Increased proliferation A pro-fibrotic phenotype Increased expression of inflammatory factors	Li et al. (144)
TNF-α	Reduced viability Increased pro-inflammatory cytokines Impaired pro-angiogenic activity	Giacomelli et al. (145)
IL-1/TNF-*α*	Decreased CFU Reduced osteogenic potentials	Zhang et al. (146)
IL-1β/TNF-α/IFN-γ/Retinol	Enhanced characteristics Enhanced initial cellular proliferation Increase in the expression of the pluripotency markers Increase in the differentiation potential Activation of the intracellular Wnt/β‐catenin pathway	Fawzy El-Sayed et al. (147)
*F. nucleatum*	Promoting cell migration and chemokine/cytokine release Inhibiting the proliferation and osteogenic differentiation	Kang et al. (148)
*Porphyromonas gingivalis-LPS*	Increased proliferation Minimal inflammatory response No effects on CFU and osteogenic potentials	Zhou et al. (149)
Antibiotics/Oral Microbiota/LPS	Inhibition of proliferation, *in vivo*, mice	Su et al. (122)

Several *in vitro* studies have shown the negative effects of inflammatory cytokines on the property and function of GMSCs. Giacomelli C et al. reported that exposure to high concentration of TNF-α reduced the viability, increased the expression of pro-inflammatory cytokines, and impaired the pro-angiogenic activity of GMSCs (145). Zhang F et al. showed that IL-1/TNF-*α* stimulation decreased CFU formation and osteogenic potentials of GMSCs (146). On the other hand, a recent study by Fawzy El-Sayed KM et al. indicated that, when cultured under a controlled low‐grade inflammatory microenvironment conferred by IL-1β/TNF-α/IFN-γ/Retinol, GMSCs displayed enhanced characteristics as presented by an increase in the initial cellular proliferation, the expression of pluripotency markers, and differentiation potentials possibly through the activation of the intracellular Wnt/β‐catenin pathway (147).

Regarding the effect of bacteria on GMSCs, Kang W et al. found that persistent exposure to *F. nucleatum* promotes cell migration and chemokine/cytokine release but suppresses the proliferation and osteogenic differentiation of GMSCs (148). *In vivo*, Su Y et al. reported that altered oral microbiome and an increased LPS level caused by antibiotic treatment led to GMSCs deficiency and proliferation inhibition and a delayed wound healing in mice (122). On the other hand, Zhou L et al. reported that *Porphyromonas gingivalis* lipopolysaccharides (Pg-LPS) increased proliferation but had no obvious effects on CFU formation and osteogenic potentials and inflammatory responses of GMSCs (149). Altogether, these studies have shown that bacteria and inflammation have various effects on the property and function of GMSCs, and further studies are necessary to clarify such effects, particularly, under the inflammatory settings *in vivo*.

## Optimizing the Property and Function of GMSCs

Even though the therapeutic and regenerative potentials of MSCs have been extensively explored in a wide range of preclinical and clinical studies, the outcomes of MSC-based therapy in clinical studies are usually not as good as observed in preclinical studies, which, in general, might be attributed to the heterogeneity of MSCs that are introduced at multiple levels (5). Up to date, numerous strategies, from the modification of culture conditions, the use of bioactive factors (e.g. cytokines, growth factors, small molecules) and harnessing the properties of biomaterials to genetic modification, have been explored to optimize the property and function of MSCs so as to improve their overall therapeutic and regenerative potentials (150, 151). Similarly, different approaches have also been explored to optimize the property and function of GMSCs (**Table 9**).

**Table 9:** Approaches for optimizing property and function of GMSCs.

Approach	Methods	Other factors	Optimized property & function		Refs
			*In vitro*	*In vivo*	
3D spheroids	Ultra-low Culture dish	Mesensphere	Stemness Adipogenic Secretome	Oral mucositis Mice	Zhang et al. (115)
	Ultra-low culture dish	Mesensphere	Stemness Trilineage and neural differentiation		Subbarayan et al. (152)
	Chitosan membrane	Spheroids	Stemness Trilineage/neural Neural differentiation		Hsu et al. (48)
	Chitosan	Spheroids	Stemness Neural Crest Chondrogenic		Hsu et al. (49)
	Microwell	Spheroids	Osteogenic		Lee et al. (153)
	Microwell	Lovastatin	Proliferation Osteogenic		Kim et al. (154)
	Microwell	FGF-4	Osteogenic		Son et al. (155)
	Ultra-low Microwell	Mesensphere Spheroids	Stemness Osteogenic Cytokine secretion		Shanbhag et al. (156)
	Floating	Neurosphere	Neural crest		Fournier et al. (43)
	Poly-L-ornithine	Defined serum-free medium	Neural crest	Rat facial nerve defect	Zhang et al. (119)
Hypoxia			Immunomodulation	Mice skin wound	Jiang et al. (60)
			Gene expression associated with neuronal development		Gugliandolo et al. (157)
Cytokines & growth factors	TNF-α		M2 macrophage	Skin wound healing Periodontitis	Nakao et al. (98)
	IL-1β		Secretomes Cell migration Anti-inflammation	Skin wound healing Epidermal substitute Engraftment	Magne et al. (95)
	FGF2	Genetic overexpression	Angiogenic factors Tube formation	In vivo angiogenesis NOD/SCID mice	Jin et al. (158)
	Sema3A	Genetic overexpression	Proliferation Osteogenic		Tian et al. (159)
Small molecules	Acetylsalicylic acid		Immunomodulation	Colitis	Yu et al. (59)
	Trichostatin A (TSA)		Anti-inflammatory Osteogenic	Rat periodontitis	Li et al. (160)
	SB431542		Osteogenic	Jawbone, minipig	Shi et al. (110)
	Cocktails		Neurogenic		Heng et al. (161)
	Ascorbic acid (AA)		Stemness Proliferation		Van Pham et al. (162)
	Ascorbic acid (AA)		Stemness Proliferation Differentiation		Fawzy El-Sayed et al. (163)
	Ascorbic acid (AA)		Osteogenic		Diomede et al. (164)
	Ascorbic acid (AA)		Osteogenic		Pizzicannella et al. (165)

### 3D Spheroids

A variety of 3D-spheroid culture methods have been applied to optimize the property and function of MSCs because 3D-spheroids allow tight cell-cell and cell-matrix interactions, thus recapitulating certain properties of the *in vivo* physicochemical microenvironment (166). Several lines of evidence have shown that 3D-spheroid culture of GMSCs can enhance their stemness, multipotency, secretomes, and therapeutic effects under different settings (**Table 9**). Early studies by Hsu S-H et al. showed that GMSCs spontaneously aggregate into 3D-spheroids with enhanced stemness, enriched neural crest cell properties, and increased trilineage and neural differentiation capacities (48, 49). For instance, our previous study showed that 3D-spheroid GMSCs formed under normal growth condition in ultra-low attachment dishes exhibit a homogenous cell morphology with a smaller cell size, an elevated expression of pluripotent genes OCT-4 and Nanog, a decreased expression of MSC associated cell surface markers, an elevated secretion of several chemokines and cytokines relevant to cell migration, survival and angiogenesis, and increased resistance to oxidative stress-induced apoptosis due to an elevated expression of manganese super-oxidative dismutase (MnSOD) (115). More importantly, systemic administration of 3D-spheroid GMSCs display enhanced therapeutic effects on chemotherapy-induced oral mucositis in mice as compared to their 2D-adherent counterparts (115). Similarly, 3D-spheroid GMSCs generated under different culture conditions also exhibit enhanced stemness, trilineage and neural differentiation potentials, and cytokine secretion (152, 156). Several studies show that GMSCs cultured in patterned microwells can aggregate into 3D-spheoids with enhanced osteogenic potentials, which are further augmented by the addition of lovastatin or FGF-4 (153–155). Under floating culture with neural culture medium, GMSCs aggregate into neurospheres with elevated expression of neural crest stem-like cells related genes and enhanced neuronal differentiation capabilities (43). Recently, our studies indicate that GMSCs cultured on poly-L-ornithine/laminin coated culture dishes with defined serum-free neural culture medium can be reprogrammed into neural crest-stem cells with enhanced differentiation capability into neuronal and glial cells (119). Meanwhile, these GMSC derived NCSC-like cells possess enhanced therapeutic effects on functional recovery and axonal regeneration in a facial nerve segmental defect model in rats (119). Altogether, these studies have demonstrated that different 3D spheroid cultures method provide efficient approaches to optimize certain properties and functions, and consequently, the therapeutic potentials of GMSCs.

### Hypoxia

Hypoxia-preconditioning represents another popularly used approach to optimize the function of MSCs (150). Jiang C et al. reported that hypoxic stimulation of GMSCs significantly promotes their immunosuppressive effects on the proliferation and activation of human peripheral blood mononuclear cells (PBMCs) *in vitro* and enhances their therapeutic effects on skin wound healing in mice (60). Most recently, a study by Gugliandolo A et al. showed that hypoxia preconditioning of GMSCs induced the expression of more genes associated with different stages of cortical development, while an increased number of genes associated with development biology and neuronal process were induced in hypoxia-preconditioned GMSCs following differentiation as compared to those cultured under normoxia (157). Meanwhile, they showed that hypoxia-preconditioned GMSCs had an increased expression of nestin, PAX6, and GAP43 upon differentiation as compared to non-preconditioned GMSCs, thus suggesting that hypoxia preconditioning enhanced the neuronal differentiation potential of GMSCs (157).

### Cytokines and Growth Factors

A panel of proinflammatory cytokines, such as TNF-1α, IL-1β, and INF-γ, and growth factors, such as HGF, TGF-β1 and FGF2, are popularly utilized to prime MSCs (150). Most recently, Magne B et al. showed that IL-1β-primed GMSCs had a different secretory profile, particularly an elevated expression of TGF-β1 and matrix metalloproteinase (MMP) pathway agonists, as compared to the naïve GMSCs (95). Functionally, IL-1β-primed GMSCs not only promoted cell migration and dermal-epidermal junction formation and suppressed inflammatory responses *in vitro*, but also significantly enhanced epidermal substitute engraftment and skin wound healing in mice after local transplantation (95). Interestingly, Nakao Y et al. recently reported that priming GMSCs by TNF-α stimulation not only increased the exosome secretion but also the exosomal expression of CD73, thus enhancing their immunomodulatory effects on polarization of anti-inflammatory M2 macrophages *in vitro (*98). *In vivo*, local application of exosomes from TNF-α primed GMSCs display significantly enhanced therapeutic effects on a mice model of periodontitis as compared to treatment with naïve GMSC-derived exosomes (98).

Using genetic approaches, Jin S et al. reported that the conditioned medium (CM) derived from human GMSCs overexpressing FGF2 (LV-FGF-2^+^-hGMSC-CM) contained a significantly increased level of not only FGF-2 but also VEGF-A and TGF-β as compared to that in naïve GMSC-CM (158). Functionally, LV-FGF-2^+^-hGMSC-CM significantly promoted chemotaxis and tube-like structure formation by HUVECs *in vitro* and angiogenesis *in vivo* as compared to naïve GMSC-CM (158). Semaphorin (Sema)-3A, a membrane-associated secreted protein, has been shown to play an important role in regulating bone remodeling (167). A recent study indicated that GMSCs overexpressing Sema3A exhibited enhanced osteogenic differentiation and proliferation capacities under the LPS-induced inflammatory environment as compared to naïve GMSCs (159). These studies suggest that utilization of certain cytokines or growth factors can prime certain properties and functions of GMSCs, thus improving their therapeutic potentials under certain conditions.

### Small Molecules

Various types of bioactive small molecules have also been explored to prime the property and function of MSCs, including GMSCs (**Table 9**). For instance, Yu T et al. recently reported that GMSCs isolated from inflammatory gingival tissues of mice (iGMSCs) exhibited a significant decline in their immunomodulatory effects on T cells *in vitro* and impaired therapeutic effects on acute colitis in mice as compared to normal tissue-derived GMSCs (59). However, pretreatment with aetylsalicylic acid (ASA) robustly rescued the impaired immunomodulatory and therapeutic effects of iGMSCs possibly through upregulating the expression of FasL in iGMSCs (59). Li Q et al. found that pretreatment with trichostatin A (TSA), an epigenetic modifier, markedly enhanced the osteogenic differentiation capacity and reduced the expression of inflammatory cytokines in GMSCs, whereas local application of TSA remarkably promoted periodontal repair in rats (160). In a recent study, Shi A et al. showed that GMSCs pretreated with SB431542, a specific TGF-β signaling inhibitor, displayed significantly increased osteogenic differentiation capacity, while local transplantation of SB431542-treated autologous pig GMSCs remarkably facilitated bone regeneration in a critical-sized mandibular bone defect model in minipig (110). Heng BC et al. showed that treatment of several types of dental MSCs, including GMSCs, with a cocktail of eight small molecules (Valproic acid, CHIR99021, Repsox, Forskolin, SP600125, GO6983, Y-27632 and Dorsomorphin) remarkably enhanced their neurogenic differentiation potentials (161). Additionally, several studies have demonstrated the use of ascorbic acid (AA) or vitamin C in priming the property of GMSCs. An early study showed that AA treatment enhanced the stemness of GMSCs as evidenced by elevated expression of several pluripotent genes such as SSEA-3, Sox-2, Oct-3/4, Nanog, and TRA-1-60 compared with control cells (162). A recent study indicated that AA stimulation also enhances GMSCs’ stemness, proliferation, and differentiation properties possibly by the activation of Wnt/*β*-catenin signaling pathways (163). Other studies have also demonstrated the pro-osteogenic effects of AA on GMSCs under different conditions (164, 165).

## Conclusion and Future Perspectives

In the last two decades, significant progress has been made in MSC-based tissue engineering (TE) and regenerative therapy across various fields of medicine, including the use of MSCs to treat a large range of human disorders in over 1200 registered and 300 completed clinical trials (https://www.clinicaltrials.gov/) (5). However, large variations exist in the therapeutic effects and overall clinical outcomes of MSC-based regenerative therapy, which might mainly be attributed to the heterogeneous properties and functions of MSCs that can be produced at different levels, such as donor characteristics, the tissue source, techniques used for cell isolation and expansion, tissue engineering, and product storage/administration (5). To date, numerous approaches have been tested to prime MSCs so as to mitigate their heterogeneity, and consequently, to improve their therapeutic effects (150). Since the initial isolation and characterization of GMSCs a decade ago (15), a growing body of studies have demonstrated their potent immunomodulatory/anti-inflammatory effects and regenerative therapeutic potentials in a variety of preclinical models of human diseases (**Figures 2** and **3**). Importantly, several lines of evidence have shown that GMSCs keep their normal karyotype and telomerase activity in long-term cultures and display no tumorigenicity following long-term inoculation into nude mice (22, 114, 162). In addition, following transplantation of human GMSCs into mouse, rat, rabbit, beagle dog, and monkey as well as in GMSC-treated animal models of autoimmune arthritis or lupus, GMSCs were well tolerated by all recipient hosts without any obvious systemic adverse effects, e.g. toxicity, abnormal immune reactions, and tumorigenesis, thus having further demonstrated the safety of GMSC transplantation and paved the way for clinical studies of GMSCs in regenerative therapy (168). Up to date, there are only two registered clinical trials on the use of GMSCs in the treatment of periodontitis (NCT03137979 and NCT03638154), but the current status and outcomes are not clear (https://www.clinicaltrials.gov/). Due to the intrinsic and extrinsic heterogeneity, there will be similar challenges for the translational application of GMSCs as to the use of other sources of MSCs. Even though certain approaches have also been tested to prime the property and function of GMSCs (**Table 9**), it is remains unknown whether such approaches can improve the therapeutic effects of GMSCs on specific disease models *in vivo*. In general, much more efforts are needed to establish the pathway for clinical trials of GMSCs, including the selection of appropriate donors, the establishment of standard operating procedures (SOP) for all steps in making GMSC products (e.g. cell isolation, expansion, storage and transportation), the use of appropriate approaches to priming GMSCs prior to administration, the identification of appropriate patient groups for treatment, etc. (5) Overall, the easy accessibility, less morbidity of harvesting, the highly proliferative activity and genomic stability, a neural crest-origin as well as the potent immunomodulatory and regenerative potentials, make GMSCs an attractive source of adult stem cells for tissue engineering and regenerative therapy.

## Author Contributions

QZ and ADL constructed the concept of the paper. DK and QZ performed the acquisition of the data and information, and mainly wrote the draft of the manuscript. AEL, QX, and ADL participated in editing and finalizing the manuscript. All authors contributed to the article and approved the submitted version.

## Funding

This work was supported by the National Institute of Dental and Craniofacial Research (NIH/NIDCR) R21DE029926-01 (ADL), the Schoenleber funding support (ADL and QZ), the Project Funding from Center for Human Appearance (CHA) at UPenn (QZ).

## Conflict of Interest

The authors declare that the research was conducted in the absence of any commercial or financial relationships that could be construed as a potential conflict of interest.
